# Transcriptome analysis of *Panax vietnamensis* var. *fuscidicus* discovers putative ocotillol-type ginsenosides biosynthesis genes and genetic markers

**DOI:** 10.1186/s12864-015-1332-8

**Published:** 2015-03-08

**Authors:** Guang-Hui Zhang, Chun-Hua Ma, Jia-Jin Zhang, Jun-Wen Chen, Qing-Yan Tang, Mu-Han He, Xiang-Zeng Xu, Ni-Hao Jiang, Sheng-Chao Yang

**Affiliations:** Yunnan Research Center on Good Agricultural Practice for Dominant Chinese Medicinal Materials, Yunnan Agricultural University, Kunming, 650201 Yunnan People’s Republic of China

**Keywords:** *Panax vietnamensis* var. *fuscidicus*, Transcriptome, Ginsenosides, Biosynthesis

## Abstract

**Background:**

*P. vietnamensis* var. *fuscidiscus*, called “Yesanqi” in Chinese, is a new variety of *P. vietnamensis*, which was first found in Jinping County, the southern part of Yunnan Province, China. Compared with other *Panax* plants, this species contains higher content of ocotillol-type saponin, majonoside R_2_. Despite the pharmacological importance of ocotillol-type saponins, little is known about their biosynthesis in plants. Hence, *P. vietnamensis* var. *fuscidiscus* is a suitable medicinal herbal plant species to study biosynthesis of ocotillol-type saponins. In addition, the available genomic information of this important herbal plant is lacking.

**Results:**

To investigate the *P. vietnamensis* var. *fuscidiscus* transcriptome, Illumina HiSeq™ 2000 sequencing platform was employed. We produced 114,703,210 clean reads, assembled into 126,758 unigenes, with an average length of 1,304 bp and N50 of 2,108 bp. Among these 126,758 unigenes, 85,214 unigenes (67.23%) were annotated based on the information available from the public databases. The transcripts encoding the known enzymes involved in triterpenoid saponins biosynthesis were identified in our Illumina dataset. A full-length cDNA of three *Squalene epoxidase* (SE) genes were obtained using reverse transcription PCR (RT-PCR) and the expression patterns of ten unigenes were analyzed by reverse transcription quantitative real-time PCR (RT-qPCR). Furthermore, 15 candidate cytochrome P450 genes and 17 candidate UDP*-glycosyltransferase* genes most likely to involve in triterpenoid saponins biosynthesis pathway were discovered from transcriptome sequencing of *P. vietnamensis* var. *fuscidiscus*. We further analyzed the data and found 21,320 simple sequence repeats (SSRs), 30 primer pairs for SSRs were randomly selected for validation of the amplification and polymorphism in 13 *P. vietnamensis* var. *fuscidiscus* accessions. Meanwhile, five major triterpene saponins in roots of *P. vietnamensis* var. *fuscidicus* were determined using high performance liquid chromatography (HPLC) and evaporative light scattering detector (ELSD).

**Conclusions:**

The genomic resources generated from *P. vietnamensis* var. *fuscidiscus* provide new insights into the identification of putative genes involved in triterpenoid saponins biosynthesis pathway. This will facilitate our understanding of the biosynthesis of triterpenoid saponins at molecular level. The SSR markers identified and developed in this study show genetic diversity for this important crop and will contribute to marker-assisted breeding for *P. vietnamensis* var. *fuscidiscus*.

**Electronic supplementary material:**

The online version of this article (doi:10.1186/s12864-015-1332-8) contains supplementary material, which is available to authorized users.

## Background

Ginsenosides are triterpenoid saponins found exclusively in *Panax* species belong to Araliaceae family. The *Panax* genus comprises approximately 14 species, more than 150 naturally occurring ginsenosides have been isolated from different parts of plants [1] and most of the saponins possess four types of aglycone moieties, i.e. protopanaxadiol, protopanaxatriol, ocotillol, and oleanolic acid types. The most widely used *Panax* species, such as *P. ginseng*, *P. quinquefolium*, and *P. notoginseng* mainly contain protopanaxadiol-type and protopanaxatriol-type saponins, the other species like *P. japonicus* and *P. zingiberensis*, contain a large amounts of oleanolic acid saponins [2,3], all of them do not have or only a small amount of ocotillol-type saponins. Up to now, only one species, *P. vietnamensis* have been found particularly accumulates surprisingly high content of ocotillol-type saponins, mainly majonoside R_2_, which is as high as 5.3% of the dried rhizome and exhibited anti-tumor and hepatocytoprotective activities [4-6].

**Figure 1:**
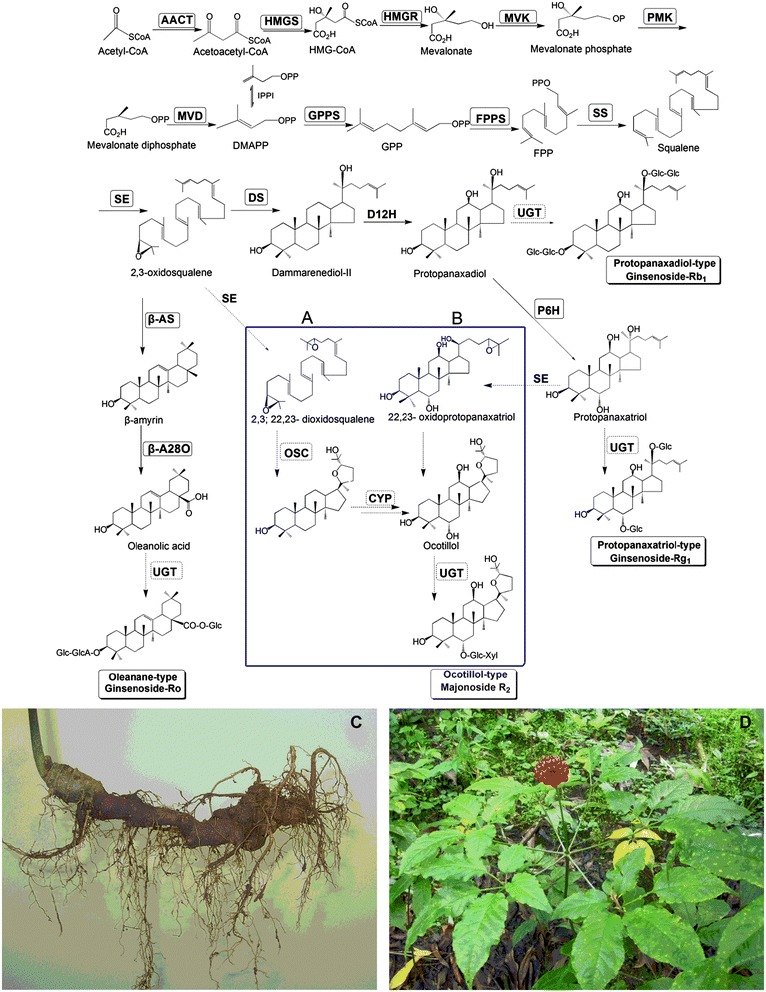
**Putative pathway for triterpene saponin biosynthesis.** Putative pathway for triterpene saponin biosynthesis in *P. vietnamensis* var. *fuscidicus*. Two proposed pathways **(A and B)** for the biosynthesis of ocotillol-type saponins, mainlymajonoside R_2_ in the horizontally grown rhizome **(C)** of *P. vietnamensis* var. *fuscidicus* **(D)**. Enzymes found in this study are boxed. Abbreviations: AACT, acetyl-CoA acetyltransferase; β-AS, β-amyrin synthase; DMAPP, dimethylallyl diphosphate; DS, dammarenediol-II synthase; FPP, farnesyl diphosphate; FPPS, farnesyl diphosphate synthase; Glc, glucose; GPP, geranyl pyrophosphate; GGPP, geranylgeranyl diphosphate; GGPPS, geranylgeranyl pyrophosphate synthase; GT, glycosyltransferase; HMG-CoA, 3-hydroxy-3-methylglutaryl coenzyme A; HMGR, HMG-CoA reductase; HMGS, HMG-CoA synthase; IPP, isopentenyl diphosphate; IPPI, IPP isomerase; MVD, mevalonate diphosphate decarboxylase; MVK, mevalonate kinase; P450, cytochrome P450; PMK, phosphomevalonate kinase; SE, squalene epoxidase; SS, squalene synthase.

2,3-oxidosqualene (OS), a precursor of terpenoids is synthesized via the mevalonic acid (MVA) pathway [7]. After the cyclization of 2,3-oxidosqalene by oxidosqualene cyclase (OSC), the triterpene skeletons are modified by hydroxylation and glycosidation that leads to the production of various ginsenosides, that are further catalyzed by cytochrome P450 monoxygenases (CYP450s) and uridine diphosphate (UDP)-dependent glycosyl-transferases (UGTs) [8] (Figure 1). The biosynthesis of protopanaxadiol, protopanaxatriol and oleanolic acid has been studied well, many genes involved in this pathway have been cloned and identified [9-17]. Recently, many putative triterpene saponin-biosynthetic genes in *Panax* species were detected using *de novo* sequencing and transcriptome analysis, especially in *P. ginseng*, *P. quinquefolius*, and *P. notoginsen*g [18-22]. Despite the pharmacological importance of ocotillol-type saponins, little is known about their biosynthesis [1]. *P. vietnamensis* is the only species found in the narrow habitat in central Vietnam with high content of ocotillol-type saponins, which is also in the list of endanger species.

There are two kinds of pathways that form ocotillol. In pathway A, ocotillol might be biosynthesized via epoxidation of the double bond at C-24-C-25 of protopanaxatriol [1]. The enzyme catalyzes this reaction could be the ortholog of squalene epoxidase (SE*)* gene, because they epoxidized the similar double bonds of squalene or protopanaxatriol (Figure 1). In pathway B, *OS* is further epoxidized to 2, 3; 22, 23- dioxidosqualene (DOS) by SE [23], followed by cyclization and hydroxylation to produce ocotillol, catalyzed by OSC and CYP450, respectively (Figure 1). In *Arabidopsis*, OSC, lupeol synthase (LUP1), directly converts DOS to oxacyclic triterpenoid epoxydammarane [23], so there might be similar OSC in *P. vietnamensis*, which catalyzed the cyclization of DOS (Figure 1).

*P. vietnamensis* var. *fuscidiscus*, called “Yesanqi” in Chinese, is a new variety of *P. vietnamensis*, which was first found in Jinping County, the southern part of Yunnan Province, China [24]. *P. vietnamensis* var. *fuscidiscus* contains a higher content of majonoside R_2_ than other genotypes of *P. vietnamensis* [2]. Therefore, *P. vietnamensis* var. *fuscidiscus* is a perfect plant species for studying the biosynthesis mechanism of ocotillol-type saponins. Interestingly, *P. vietnamensis* var. *fuscidiscus* was also found in Yuanyang and Lvchun County, Honghe prefecture of Yunnan Province and some of them are found for more than 15 years and exhibited remarkable disease resistance under the high temperature and rainy conditions in this district, suggested that this specie could be used to improve disease resistance of *P. notoginseng*, an important cultivated *Panax* species in Yunnan Province of China.

Our goal of this study is to characterize the transcriptome of *P. vietnamensis* var. *fuscidiscus* using Illumina HiSeq™ 2000 sequencing platform, to discover the candidate genes that encode enzymes in the triterpene saponin biosynthetic pathway, especially in ocotillol-type saponins biosynthesis, and produce information on SSR markers to facilitate the marker-assisted breeding of this species.

## Results and discussion

### Illumina sequencing and *de novo* assembly

*P. vietnamensis* var. *fuscidiscus* root tissue was used for transcriptome sequencing and analysis because root organs have been used for medicinal purpose. A cDNA library was constructed from total RNA of *P. vietnamensis* var. *fuscidiscus* roots, and sequenced using Illumina paired-end sequencing technology. After removal of adaptor sequences, ambiguous reads and low-quality reads (Q20 < 20), a total of 114,703,210 clean reads were obtained. The Q20 percentage (sequencing error rate < 1%) and GC percentage were 97.23% and 43.25%, respectively. An overview of the sequencing and assembly statistics are shown in Table 1. The high quality reads obtained in this study have been deposited in the NCBI SRA database (accession number: SRA146484).

**Table 1:** **Summary of Illumina Paired-end sequencing and assembly for** ***P. vienamensis*** **var.** ***fuscidiscus***

**Database**	**Number**	**Total length(bp)**
Total Clean reads	114,703,210	11,470,321,000
Q20 percentage	97.23%	
GC percentage	43.25%	
Number of contigs	161,443	218,944,221
Average length of contigs (bp)	1,356	
Max length of contigs (bp)	15,880	
Min length of contigs (bp)	201	
Contig size N50 (bp)	2,087	
Number of unigenes	126,758	165,291,103
Average length of unigenes (bp)	1,304	
Max length of unigenes (bp)	15,896	
Min length of unigenes (bp)	201	
Unigene size N50 (bp)	2,108	

**Figure 2:**
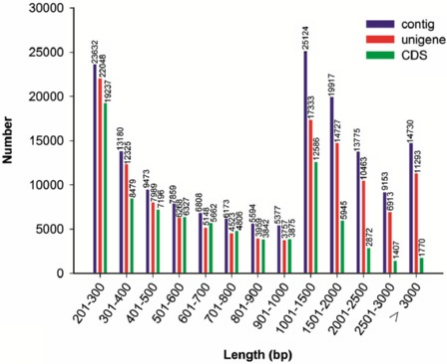
**The length distribution of contigs, unigenes and CDSs.** Overview of the *P. vietnamensis* var. *fuscidiscus* transcriptome assembly and the length distribution of the CDS.

All the clean reads (114,703,210) were *de novo* assembled using the Trinity program into 161,443 contigs consisting of 218,944,221 bp. The size of the contigs ranged from 201 to 15,880 bp, with a mean length of 1,356 bp and N50 length of 2,087 bp. Among these contigs, 82,699 (51.23%) were longer than 1000 bp, and 46,915 (29.06%) contigs were shorter than 500 bp. Using paired-end joining and gap-filling methods, these contigs were further assembled into126,758 unigenes with an average length of 1,304 bp and an N50 length of 2,108 bp. There were 60,741 unigenes (47.92%) longer than 1,000 bp, and 28,676 unigenes (22.62%) longer than 2,000 bp (Figure 2). In this study, the coding sequences (CDS) from all *P. vietnamensis* var. *fuscidiscus* unigene sequences were also detected and a total of 84,004 CDSs were obtained, among them, 24,580 CDSs (29.26%) were longer than 1,000 bp (Figure 2).

To evaluate the quality of the assembled unigenes, all the usable sequencing reads were realigned to the unigenes using SOAPaligner [25], with up to 2 mismatches allowed. The sequencing depth ranged from 0.0173 to 50,534 fold, with an average of 50.67 fold. About 86.73% of the unigenes were realigned by more than 10 reads, 31.60% were supported by more than 100 reads, and 7.73% were supported by more than 1,000 reads (Additional file 1). In order to assess the extent of transcript coverage provided by unigenes and to evaluate how coverage depth affected the assembly of unigenes, we plotted the ratio of assembled unigene length to *P. notoginseng* orthologs length against coverage depth (Additional file 2A). Although many of the deeply sequenced *P. vietnamensis* var. *fuscidiscus* unigenes failed to cover the complete coding regions of their *P. notoginseng* orthologs, most of *P. notoginseng* orthologs coding region can be covered by corresponding unigenes. To certain extent, increased coverage depth can result in higher coverage of the coding regions. The percentage of *P. notoginseng* orthologs coding sequence covered by all *P. vietnamensis* var. *fuscidiscus* unigenes was also performed. We found that 14,892 of the orthologs were covered by with a percentage of more than 80% and 3,252 of the orthologs were covered by unigenes with a percentage from 40% to 80%. Furthermore, 326 orthologs were covered with only 20% or lower (Additional file 2B).

Due to the lack of *P. vietnamensis* var. *fuscidiscus* reference geneome availability, the reads produced by Illumina HiSeq™2000 were assembled using the *de novo* assembler Trinity. In this study, the assembly results indicated that the length distribution pattern and mean length of contigs and unigenes was similar to those in the previous Illumina-transcriptome studies [26-28], suggesting that the transcriptome sequencing data from *P. vietnamensis* var. *fuscidiscus* are assembled well. Compared to previous transcriptomic studies in *Panax* species [18-22], we produced more numbers of unigenes, indicating that *P. vietnamensis* var. *fuscidiscus* genome is gene rich in comparison to *Panax* species.

### Functional annotation

A total of 85,214 unigenes (67.23%) were annotated based on the information available from public databases including NCBI non-redundant protein (Nr), Swiss-Prot protein, Cluster of Orthologous Groups (COG), and the Kyoto Encyclopedia of Genes and Genomes (KEGG) (Table 2). Among them, 16,602 unigenes showed significant matches to all four databases. Unigenes that were annotated as unique in public databases are as follows: 16,097 unigenes in the Nr database, 157 unigenes in the SwissProt database, 1 unigenes in the COG database, and 67 unigenes in the KEGG database (Additional file 3). Furthermore, about 32.77% of unigenes (41,544) did not show any matches to known genes, these remaining unaligned unigenes may be considered as novel transcripts and specific genes from *P. vietnamensis* var. *fuscidiscus*.

**Table 2:** **Summary of the annotation percentage of** ***P. vienamensis*** **var.** ***fuscidiscus*** **as compared to public database**

**Database**	**Number of unigenes**	**Annotation percentage (%)**
Nr	84,983	67.04
SwissProt	66,471	52.44
KEGG	26,730	21.09
COG	34,918	27.55
All annotated unigenes	85,214	67.23
Total unigenes	126,758	

Our results showed that approximately 95% of unigenes over 1,000 bp in length had BLAST matches against the Nr database, whereas only 41% of unigenes with lengths shorter than 1,000 bp generated BLAST matches (Additional file 4A). The same tendency was also observed in BLAST results against the SwissProt database (Additional file 4B). The e-value distribution of the top hits in the Nr database revealed that 62.34% of the mapped unigenes showed significant homology (e-value < 10^−50^), and 21.35% unigenes had high similarity (greater than 80%) (Additional file 5A and C). The e-value and similarity distributions of the top hits in the Swiss-Prot database had a comparable pattern with 47.51% and 13.48% of the sequences possessing significant homology and similarity, respectively (Additional file 5B and D). Our results also showed that 39.04% of the unigenes showed significant homology with gene sequences from *Vitis vinifera* (9,011, 19.07%), followed by *Arabidopsis thaliana* (11.60%), *Glycine max* (10.97%), and *Medicago truncatula* (9.14%) (Additional file 6).

### Gene ontology classification

**Figure 3:**
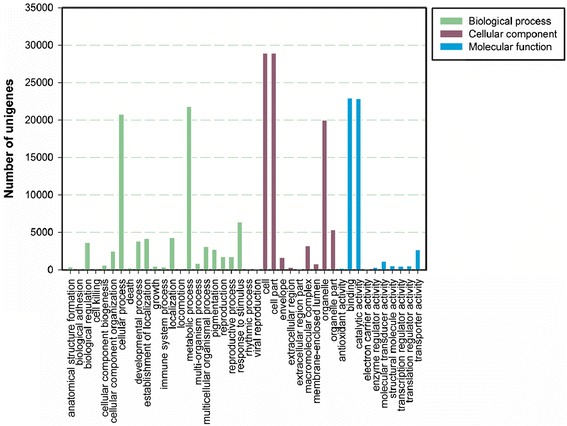
**Gene Ontology classification of assembled unigenes.** The unigenes were categorized into three main categories biological process, cellular component and molecular function.

Based on the Nr annotation, Gene Ontology (GO) classification was used to classify the functions of all unigenes. A total of 43,163 unigenes were assigned to one or more gene ontology categories, 88,984 unigenes were from the cellular component, 79,483 unigenes from the biological process, and 51,542 unigenes from the molecular function. Under the biological process category: GO classification belongs to metabolic process (21,803, 27.43%), cellular process (20,747, 26.10%), and response to stimulus (6,352, 7.99%). In the cellular component group, unique sequences related to cell (28,886, 32.46%), cell part (28,886, 32.46%), organelle (19,977, 22.45%), and organelle part (5,339, 6.00%) were found. For the molecular function category, binding (22,925, 44.48%) and catalytic activity (22.836, 44.30%) represented the majority of unique sequences (Figure 3; Additional file 7).

### Conserved domain annotation and COG classification

The conserved domains/families of the assembled unigenes encoding proteins were searched against the Pfam database (version 26.0) using Pfam_Scan program. A total of 3,602 conserved domains/families were identified from 46,649 unigenes (36,80% of all unigenes) (Additional file 8). Among these protein domains/families, pentatricopeptide repeat domain (PPR) is the most abundant domain type, found in 2,822 unigenes. The PPR containing proteins are commonly found in the plants and although its function is still unclear, the PPR domain has been found in proteins involved in RNA editing in a number of recent studies [29-32]. Other highly represented domains/families were, WD repeat (2,520 unigenes), Protein kinase domain (2,468 unigenes), and Leucine Rich Repeat (2,449 unigenes). The WD repeat and Leucine Rich Repeat are involved in protein-protein interactions [33,34]. The role of protein kinase domain is found in signal transduction pathways, development, cell division, and metabolism in higher organisms [35,36]. Other domains identified abundantly included PPR repeat family (2,354 unigenes), RNA recognition motif (1,188 unigenes), Protein tyrosine kinase (1,035 unigenes), ABC transporter (488 unigenes), Mitochondrial carrier protein (462 unigenes), and Myb-like DNA-binding domain (459 unigenes). For perspective, we have listed the top 20 most abundant protein domains/families in (Additional file 9).

**Figure 4:**
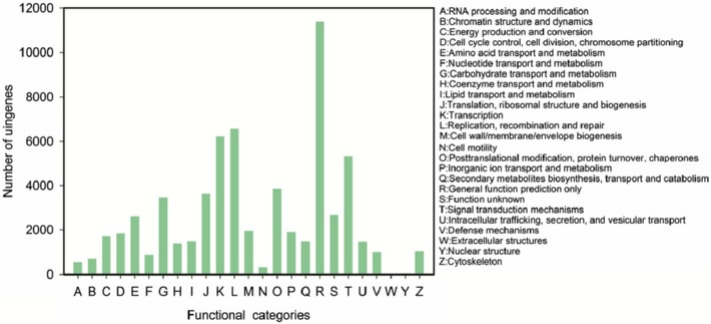
**COG function classification of** ***P. vienamensis*** **var.** ***fuscidiscus*** **.**

All unigenes were subjected to a search against the COG database for functional prediction and classification. In total, 34,918 unigenes were annotated and grouped into 25 COG classifications. However, some of these unigenes were assigned to multiple COG classifications, altogether 63,521 COG functional annotations were obtained. Among the 25 COG categories, the cluster for general function prediction was the largest group (11,382, 17.92%), followed by replication, recombination and repair (6,561, 10.33%), transcription (6,223, 9.80%), and signal transduction mechanisms (5,338, 8.40%) (Figure 4).

### Functional classification by KEGG

**Figure 5:**
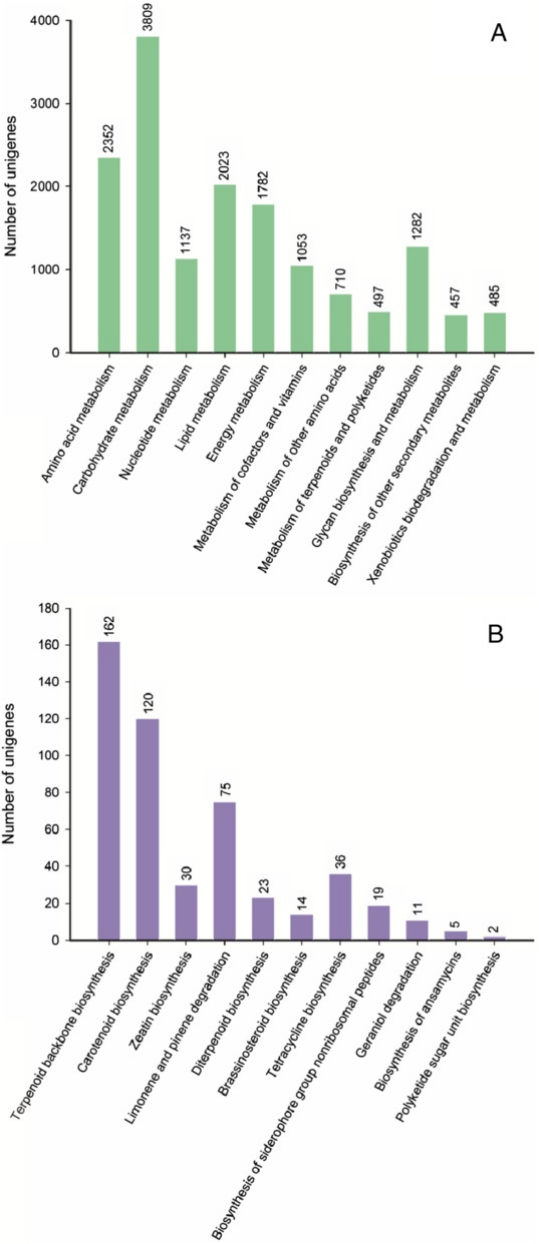
**Pathway assignment based on KEGG. (A)** Classification based on metabolism categories; **(B)** Classification based on metabolism of terpenoids and polyketides.

To elucidate the active biochemical pathways in *P. vietnamensis* var. *fuscidiscus*, unigenes were compared against the KEGG using BLASTx with an e-value < 1e^−10^ and the corresponding pathways were established. KEGG pathway analysis is helpful for predicting potential genes and their functions at a whole transcriptome level. A total of 26,730 unigenes (21.09%) were annotated with KEGG and were assigned to 269 KEGG pathways (Additional file 10). RNA transport had the largest number of unigenes (825), followed by spliceosome (812 unigenes), protein processing in endoplasmic reticulum (697 unigenes), plant hormone signal transduction (684 unigenes), ubiquitin mediated proteolysis (679 unigenes), glycolysis/gluconeogenesis (636 unigenes), and purine metabolism (616 unigenes). KEGG metabolic pathways presented in our dataset include carbohydrate metabolism (3,809 unigenes), amino acid metabolism (2,352 unigenes), nucleotide metabolism (1,137 unigenes), lipid metabolism (2,023 unigenes), energy metabolism (1,782 unigenes), glycan biosynthesis and metabolism (1,282 unigenes), metabolism of cofactors and vitamins (1,053 unigenes), metabolism of other amino acids (710 unigenes), metabolism of terpenoids and polyketides (497 unigenes), biosynthesis of other secondary metabolites (457 unigenes), and xenobiotics biodegradation and metabolism (485 unigenes) (Figure 5A). In the metabolism of terpenoids and polyketides category, the most represented subcategories were terpenoid backbone biosynthesis (162 unigenes), followed by carotenoid biosynthesis (120 unigenes), limonene and pinene degradation (75 unigenes), tetracycline biosynthesis (36 unigenes), zeatin biosynthesis (30 unigenes), diterpenoid biosynthesis (23 unigenes), siderophore group nonribosomal peptides (19 unigenes), brassinosteroid biosynthesis (14 unigenes), geraniol degradation (11 unigenes), ansamycins biosynthesis (5 unigenes), and polyketide sugar unit biosynthesis (2 unigenes) (Figure 5B). These annotations will be a valuable resource for further research on specific pathways, structures and functions of genes in *P. vietnamensis* var. *fuscidiscus*.

### SSR marker discovery

The potential SSRs were detected in all of the 126,758 assembled unigenes using MISA software. A total of 21,320 SSRs were identified in 17,780 unigenes (Table 3). Of all the SSR containing unigenes, 2,918 sequences contained more than one SSR, and 1,207 SSRs were present in compound form. The information of SSRs derived from all unigenes is shown in Additional file 11. Among these SSRs, the most frequent repeat motifs were di-nucleotides (11,197, 52.53%), followed by tri-nucleotides (5,987, 28.08%), tetra-nucleotides (2,352, 11.03%), penta-nucleotides (924, 4.33%), and hexa-nucleotides (860, 4.03%). Among the SSR with tandem repeats, SSRs with six tandem repeats (5,657, 26.53%) were most prevalent, followed by five tandem repeats (3,988, 18.71%), seven tandem repeats (3,466, 16.26%), and four tandem repeats (3,356, 15.74%) (Table 4). The di-nucleotide repeat AG/CT (26.7%) was the most common motif, followed by the motif AT/AT (19.6%), AAG/CTT (7.5%), and AC/GT (6.2%). Our findings indicated that unigenes containing SSR markers were abundant in *P. vietnamensis* var. *fuscidiscus*. Based on those SSRs, 39,336 primer pairs were successfully designed using Primer3 (Additional file 12). The unigene derived markers generated in this study represent a valuable genetic resource for SSR mining and will aid future applications in research of this important herb crop.

**Table 3:** **Summary of SSR searching results**

**Item**	**Number**
Total number of sequences examined	126,758
Total size of examined sequences (bp)	165,291,103
Total number of identified SSRs	21,320
Number of SSR containing Sequences	17,780
Average number of SSRs per 10 kb	1.29
Number of sequences containing more than 1 SSR	2,918
Number of SSRs present in compound formation	1,207

**Table 4:** **Distribution of identified SSRs using the MISA software**

**Motif**	**Repeat numbers**									**Total**	**%**
	**4**	**5**	**6**	**7**	**8**	**9**	**10**	**11**	**≥11**		
Di-	0	0	4,030	2,480	1,576	1,240	1,230	627	14	11,197	52.52
Tri-	0	3,433	1,448	966	126	2	0	3	9	5,987	28.08
Tetra-	1,789	446	96	12	0	6	0	0	3	2,352	11.03
Penta-	812	94	10	7	0	0	0	0	1	924	4.33
Hexa-	755	15	73	1	9	0	4	2	1	860	4.03
Total	3,356	3,988	5,657	3,466	1,711	1,248	1,234	632	28	21,320	100
%	15.74	18.71	26.53	16.26	8.03	5.85	5.79	2.96	0.13	100	

### Validation of SSR markers

Thirty SSR primer pairs were randomly selected and synthesized to evaluate the amplification efficiency and polymorphism in 13 *P. vietnamensis* var. *fuscidiscus* accessions from different countries and different genetic backgrounds. Twenty-nine (96.67%) of the primer pairs successfully amplified clear and repeatable bands. Among the 29 successful primer pairs, 24 (80.00%) primer pairs produced PCR amplicons at the expected size, and 4 (13.33%) primer pairs generated PCR fragments longer than expected. We also found 15 (50.00%) primer pairs exhibited polymorphisms (Additional file 13) among the 13 *P. vietnamensis* var. *fuscidiscus* accessions. The observed number of alleles (*No*) ranged from 0.25 to 0.52, with an average value of 0.42; the effective number of alleles (*Ne*) ranged from 0.20 to 0.41, with a mean level of 0.34; Shannon’s information index (*I*) varied from 0.16 to 0.33, with an average value of 0.27; the number of polymorphic loci (*NP*) ranged from 0.80 to 4.40, with a mean level of 2.5; the percentage of polymorphic loci (*PPB*) ranged from 8.89 to 48.89, with a mean level of 28.15; and polymorphism information content (*PIC*) values ranged from 0.29 to 0.50 with an average of 0.44. These results indicated that there is a good genetic diversity existed among 13 *P. vietnamensis* var. *fuscidiscus* accessions.

**Figure 6:**
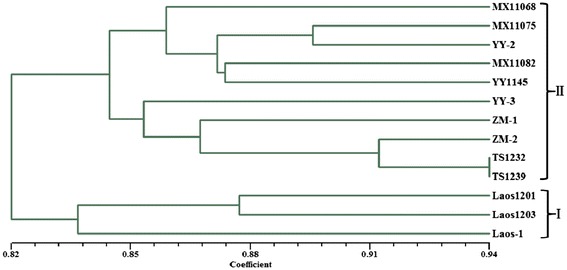
**UPGMA dendrogram of 13 accessions of** ***P. vienamensis*** **var.** ***fuscidiscus.*** Dendrogram constructed with UPGMA clustering method among 13 different accessions of *P. vienamensis* var. *fuscidiscus*.

The dendrogram constructed based on UPGMA (un-weighted pair group method with arithmetic average) clustering method was used to perform genetic correlation analysis among the 13 *P. vietnamensis* var. *fuscidiscus* accessions (Figure 6). The coefficients of genetic similarity among the 13 *P. vietnamensis* var. *fuscidiscus* accessions ranged from 0.82 to 0.94, indicating a high genetic similarity among them. UPGMA cluster analysis grouped these individuals into two groups at the similarity level of 0.836. According to the dendrogram, all the 3 accessions from Laos were clustered into cluster I. In cluster II, all the 10 accession of *P. vietnamensis* var. *fuscidiscus* from China were clustered into one group. The results of the cluster analysis showed that the individuals from the same area tend to clustered together. Therefore, the UPGMA cluster analysis based on SSR data was closely related to the geographical origins. Meanwhile, these results demonstrate that SSRs primer pairs derived from *P. vietnamensis* var. *fuscidiscus* unigenes can distinguish varieties without morphological diversities, and will be a powerful tool for genetic applications in this herb crop.

### Candidate genes encoding enzymes involved in ginsenosides biosynthesis

The transcripts encoding all the known enzymes involved in triterpenoid saponin pathway were discovered from this Illumina transcriptome dataset, including AACT (acetyl-CoA acetyltransferase), HMGS (HMG-CoA synthase), HMGR (HMG-CoA reductase), MVK (mevalonate kinase), PMK (phosphomevalonate kinase), MVD (mevalonate diphosphate decarboxylase), GGPPS (geranylgeranyl pyrophosphate synthase), FPPS (farnesyl diphosphate synthase), IPPI (isopentenyl diphosphate isomerase), SS (squalene synthase), SE, β-AS (β-amyrin synthase), DS (dammarenediol-II synthase), D12H (dammarenediol 12-hydroxylase), P6H (protopanaxadiol 6-hydroxylase), and β-A28O (β-amyrin 28-oxidase) (Table 5, Additional file 14). The discovery of many genes related to triterpenoid pathway may help us to investigate the cause of high content of protopanaxadiol-type and protopanaxatriol-type saponins, such as Rb1, Rd and Rg_1_ in *P. vietnamensis* var. *fuscidiscus*.

**Table 5:** **Transcripts involved in triterpene saponin biosynthesis in** ***P. vietnamensis*** **var.** ***fuscidiscus***

**Gene name**	**EC number**	**Unigene number**
AACT, acetyl-CoA acetyltransferase	2.3.1.9	8
HMGS, hydroxymethylglutaryl-CoA synthase	2.3.3.10	7
HMGR, hydroxymethylglutaryl-CoA reductase	1.1.1.34	4
MVK, mevalonate kinase	2.7.1.36	3
PMK, phosphomevalonate kinase	2.7.4.2	10
MVD, mevalonate diphosphate decarboxylase	4.1.1.33	2
GGPPS, geranylgeranyl pyrophosphate synthase	2.5.1.29	64
FPPS, farnesyl diphosphate synthase	2.5.1.10	34
IPPI, isopentenyl diphospate isomerase	5.3.3.2	1
SS, squalene synthase	2.5.1.21	6
SE, squalene epoxidase	1.14.99.7	15
DS, dammarenediol-II synthase	4.2.1.125	1
β-AS, β-amyrin synthase	5.4.99.39	11
β-A28O, β-amyrin 28-oxidase (CYP716A52v2 in *P. ginseng*)	1.14.13.-	1
D12H, dammarenediol 12-hydroxylase (CYP716A47 in *P. ginseng*)	1.14.13.183	4
P6H, protopanaxadiol 6-hydroxylase (CYP716A53v2 in *P. ginseng*)	1.14.13.184	1

Majonoside R_2_ is the main ginsenoside in *P. vietnamensis* var. *fuscidiscus*, so we focused on the discovery of the putative genes that might be involved in ocotillol-type ginsenoside biosynthesis. As mentioned above, the formation of ocotillol needs SE and OSC with “new” functions. Generally, SE catalyzes the epoxidation of squalene to OS in terpenoid biosynthesis, but in *P. vietnamensis* var. *fuscidicus*, SE might catalyze the epoxidation of terminal olefin of protpanaxatriol or OS (Figure 1). Moreover, 15 unigenes matched to SE of other plants were discovered in our Illumina dataset. Using the primers designed based on the sequences of these SE unigenes, a full-length cDNA of three SE genes were obtained using reverse transcription PCR (RT-PCR), named PvfSE1, PvfSE2, and PvfSE3, respectively (GenBank: KJ946467, KJ946468 and KJ946469). The three cloned *SE* genes may play different roles in sterol or ginsenoside biosynthesis in this new variant (data not shown), and a series of relevant studies are currently underway to determine the function of them. Many genes encoding OSCs have been isolated in plants; including those encode β-AS, DS, lupeol synthase (LUS), and cycloartenol synthase (CAS) [16,37-39]. Except for β-AS and DS, no unigene matched to LUS and CAS was found (Table 5). Characterizing the functions of these unigenes will help us for understanding the molecular mechanism of biosynthesis of ocotillol-type ginsenoside.

### The cytochrome P450 monooxygenases and UDP-glycosyltransferase genes

Identification of specific CYP450 enzymes responsible for the production of particular metabolites is difficult due to its large numbers [40]. However, only a few CYP450s have been identified in plants, which involved in triterpenoid saponins biosynthesis. The CYP716A subfamily members in *M. truncatula* (CYP716A12) and *V. vinifera* (CYP716A15 and CYP716A17) are multifunctional oxidases, with β-A28O, α-amyrin 28-oxidase and lupeol 28-oxidase activities [41,42]. In *P. ginseng*, three CYP716A subfamily members have been isolated and characterized functionally, encode P6H (CYP716A47), D12H (CYP716A53v2) and β-A28O (CYP716A52v), respectively [13-15]. Licorice (*Glycyrrhiza uralensis*) CYP88D6 catalyze C-11 oxidation of β-amyrin in glycyrrhizin biosynthesis [43], while GuCYP72A154 and *M. truncatula* CYP72A63 catalyze C-30 oxidation of β-amyrin [44]. Both *G. max* CYP93E1, licorice CYP93E2 and CYP93E3 catalyze the C-24 hydroxylation of β-amyrin and sophoradiol in soyasaponin biosynthesis [43,45,46]. Oat (*Avena strigosa*) CYP51H10 is able to catalyze both hydroxylation and epoxidation of β-amyrin to produce 12, 13β-epoxy-3β, 16β-dihydroxy-oleanane [47-49]. Arabidopsis CYP708A2 and CYP705A5 were identified as a thalinol hydroxylase and thaliana-diol desaturase, respectively [50]. *M. truncatula* CYP72A61v2 and CYP72A68v2 catalyze C-22 of 24-OH-β-amyrin and C-23 of oleanolic acid, respectively [46].

**Figure 7:**
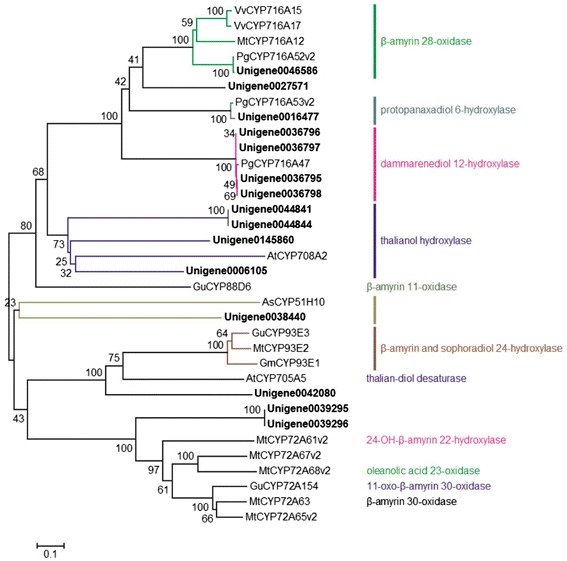
**Phylogenetic tree of CYP450s.** Phylogenetic tree of the *P. vienamensis* var. *fuscidiscus* CYP450s. Phylogenetic tree constructed based on the deduced amino acid sequences for the *P. vienamensis var. fuscidiscus* CYP450s (bold letters) and other plant CYP450s involved in triterpenoid biosynthesis. Protein sequences were retrieved from NCBI GenBank using the following accession numbers: *Vitis vinifera* VvCYP716A15, (BAJ84106.1) and VvCYP716A17 (BAJ84107.1); *Medicago truncatula* MtCYP716A12, (ABC59076.1), MtCYP93E2 (ABC59085), MtCYP72A63 (H1A981.1), MtCYP72A65v2, (BAL45202), MtCYP72A67v2 (BAL45203) and MtCYP72A68v2 (BAL45204), and MtCYP72A61v2 (BAL45199); *Panax ginseng* PgCYP716A52v2 (AFO63032.1), PgCYP716A53v2 (I7CT85.1) and PgCYP716A47 (H2DH16.2); *Arabidopsis thaliana* AtCYP708A2 **(**NP_001078732.1) and AtCYP705A5 (EFH40098); *Glycyrrhiza uralensis* GuCYP88D6 (B5BSX1.1), GuCYP93E3 (BAG68930) and GuCYP72A154 (H1A988.1); *Avena strigosa* AsCYP51H10 (ABG88965.1); *Glycine max* GmCYP93E1 (NP_001236154.1).

For discovering the candidate CYP450s involved in ginsenosides biosynthesis in the transcriptomic data of *P. vietnamensis* var. *fuscidicus*, 251 unigenes which is annotated to be CYP450 (Additional file 15) were compared with CYP450s mentioned above. As shown in Figure 7, the orthologous genes of PgCYP716A52v2 (unigene 0046586), PgCYP716A53v2 (unigene0016477), and PgCYP716A47 (unigene0036796, unigene0036797, unigene0036795, and unigene0036798) were found. Besides, one unigene (unigene0027571) is also belong to CYP716A subfamily, indicate this unigene may has different functions from other CYP716A subfamily in *P. ginseng*. Furthermore, 4 unigenes (unigene0044841, unigene0044844, unigene0145860, and unigene0006105) are highly homologous to *A. thaliana* thalianol hydroxylase (AtCYP708A2). Unigene0038 is homologous to AsCYP51H10, unigene0042080 is homologous to GuCYP93E3 and AtCYP705A5, unigene0039295 and unigene0039296 are homologous to MtCYP72A67v2 and MtCYP72A8v2 (Figure 7). Two unigenes (unigene0039295 and unigene0039296) are the orthologous gene of 11-oxo-β-amyrin 30-oxidase and highly homologous to GuCYP72A154, MtCYP72A63, MtCYP72A61v2, and MtCYP72A68v2 [44,46]. Unigene00402080 is highly homologous to GuCYP93E3 and MtCYP93E2 [43,51], may encode enzyme catalyze C-24 hydroxylation of β-amyrin.

**Figure 8:**
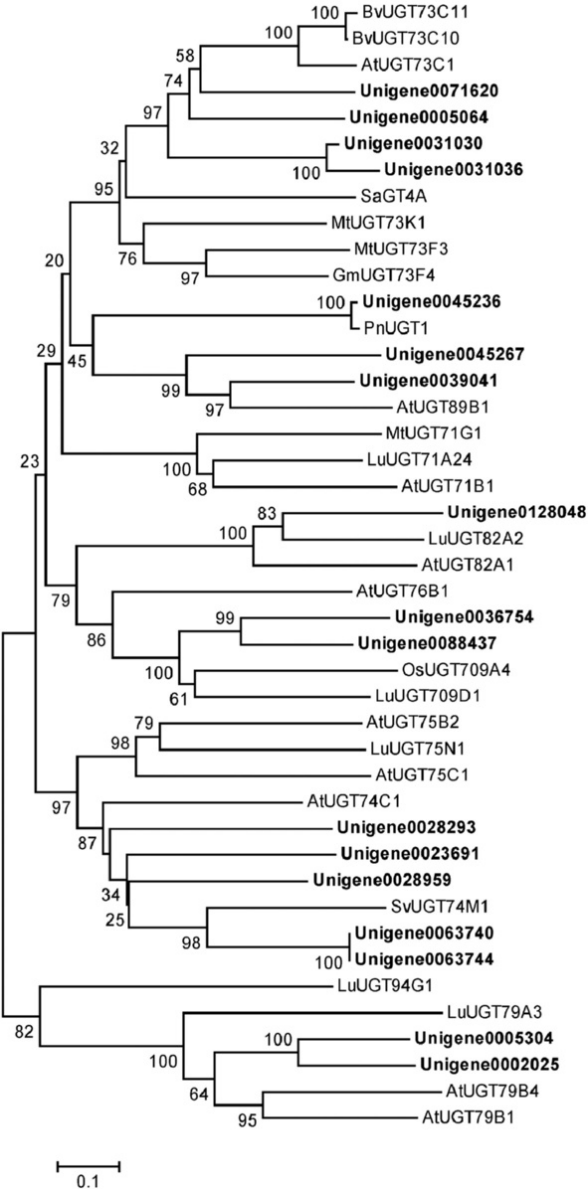
**Phylogenetic tree of UGTs.** Phylogenetic tree constructed based on the deduced amino acid sequences for the *P. vienamensis* var. *fuscidiscus* UGTs (bold letters) and other plant UGTs. Accession numbers in the NCBI GenBank database are as follows: *Barbarea vulgaris* BvUGT73C11 (AFN26667) and BvUGT73C10 (AFN26666); *Arabidopsis thaliana* AtUGT73C1 (NP_181213.1), AtUGT82A1 (NP_188864.1), AtUGT76B1 (NP_187742.1), AtUGT71B1 (NP_188812.1), AtUGT89B1 (NP_177529.2), AtUGT75B2 (NP_172044.1), AtUGT75C1 (NP_193146.1), AtUGT74C1 (NP_180738.1), AtUGT79B4 (Q9LJA6.1) and AtUGT79B1 (Q9LVW3.1); *Solanum aculeatissimum* SaGT4A (BAD89042); *Medicago truncatula* MtUGT73K1 (AAW56091), MtUGT73F3 (ACT34898) and MtUGT71G1 (AAW56092); *Glycine max* GmUGT73F4 (BAM29363); *Panax notoginseng* PnUGT1 (JX018210); *Oryza sativa* OsUGT709A4 (Q7XHR3); *Saponaria vaccaria* SvUGT74M1 (ABK76266); *Linum usitatissimum* LuUGT71A24 (AFJ52909), LuUGT82A2 (AFJ52979), LuUGT709D1 (AFJ53007), LuUGT75N1 (AFJ52962), LuUGT94G1 (AFJ53037.1), LuUGT79A3 (AFJ52973.1).

UGTs catalyze the glucosylation of C-3, C-12, C-20 hydroxyl, and C28-carboxyl for the biosynthesis of ginsenosides in *P. vietnamensis* var. *fuscidicus*. Even though UGTs catalyze the last committed step of ginsenoside biosynthesis; no UGT was functionally characterized from *Panax* species, only one putative UGT gene (PnUGT1) was cloned from *P. notoginseng* [52], which had relative close relationship to the triterpene UDP-glucosyltransferase of *M. truncatula* UGT71G1 [53]. In cDNA library of *P. vietnamensis* var. *fuscidicus*, 282 unigenes were found to encode UGTs (Additional file 16). The phylogenetic relationship between UGTs from *P. vietnamensis* var. *fuscidicus* and characterized UGTs from other plants was depicted in Figure 8. Except the orthologous genes of *PnUGT1* (unigene0045236), unigene0071620 is highly homologous to *Barbarea vulgaris* UGT73C11 and UGT73C10, which catalyze sapogenin 3-*O*-glucosylation [54], suggested that unigene0071620 has the same function in *P. vietnamensis* var. *fuscidicus*. Besides, unigene005064, unigene0031030, and unigene0031036 have close relationship to *Solanum aculeatissimum* steroidal saponin UDP-glucosyltransferase SaGT4A [55], *M. truncatula* UGT73F3 [56], MtUGT73K1, MtUGT71G1 [53], and soybean UGT73F4 [57], indicated that those unigenes are also involved in ginsenoside biosynthesis. Furthermore, 2 unigenes (unigene0063740 and unigene0063744) have close relationship to *Saponaria vaccaria* UGT74M1, which is a triterpene carboxylic acid glucosyltransferase [58], suggested that these unigenes may catalyze the glucosylation of C28-carboxyl for the biosynthesis of ginsenoside Ro.

### RT-qPCR analysis of the ginsenoside synthesis related genes

**Figure 9:**
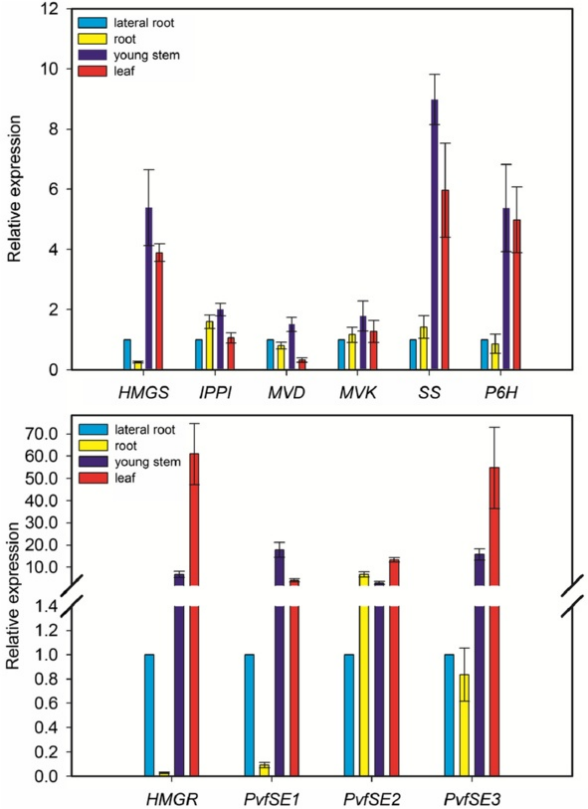
**qRT-PCR analysis of unigenes involved in triterpene saponin biosynthesis.** Validation of candidate *P. vietnamensis* var. *fuscidiscus* unigenes involved in triterpene saponin biosynthesis by qRT-PCR. Bars represent the mean (± SD) of four experiments.

The RT-qPCR analysis was used to investigate the tissue-specific expression patterns of 10 unigenes related to ginsenoside biosynthesis in this species. The expression pattern of these genes is shown in Figure 9. The unigenes encoding HMGS, MVK, MVD, and IPPI were expressed at much higher level in young stems than in other tissues (lateral roots, root and leaves). The gene encoding SS was highly expressed in the leaves and stems. The HMGR gene showed very high expression in the leaf tissue. All genes mentioned above play a role in upstream biochemical reactions of the ginsenoside pathway, and showed high expression in leaves and young stems, which indicates that leaves and young stems are the main factories for synthesizing the precursors of ginsenosides. SE gene was involved in the formation of 2,3-oxidosqualene, a precursor of various ginsenosides. To further identify the potential candidates from SE homologs involved in ginsenoside biosynthesis, the expression levels of three putative SE genes (SE1, SE2, and SE3) in different organs were analyzed. SE1 and SE3 genes were expressed much higher in young stems and leaves than in other tissues, respectively (Figure 9). Whereas the expression level of SE2 was higher in the roots as compared to that of SE1 and SE3. These three putative *SE* genes have different expression patterns in different tissues, similar to what was found in previous studies [59]. Thus, we supposed that these three putative SE genes play different roles in ginsenosides biosynthesis. The gene encoding P6H was highly expressed in leaves and young stems than in roots and hairy roots. The P6H was predicted to catalyze protopanaxadiol to protopanaxatriol. A higher expression of P6H observed in leaves and young stems but protopanaxatriol-type ginsenosides accumulated mainly in roots and hairy roots, again indicating that leaves and young stems were the main synthesis site of the triterpene skeletons. The results demonstrate that several genes involved in ginsenoside biosynthesis showed diverse expression patterns in different tissues. The analysis of the expression patterns of these genes in different tissues will be helpful to further understand the mechanism of ginsenoside biosynthesis.

### Quantitative analysis of five major triterpene saponins in roots of *P. vietnamensis* var. *fuscidicus*

**Figure 10:**
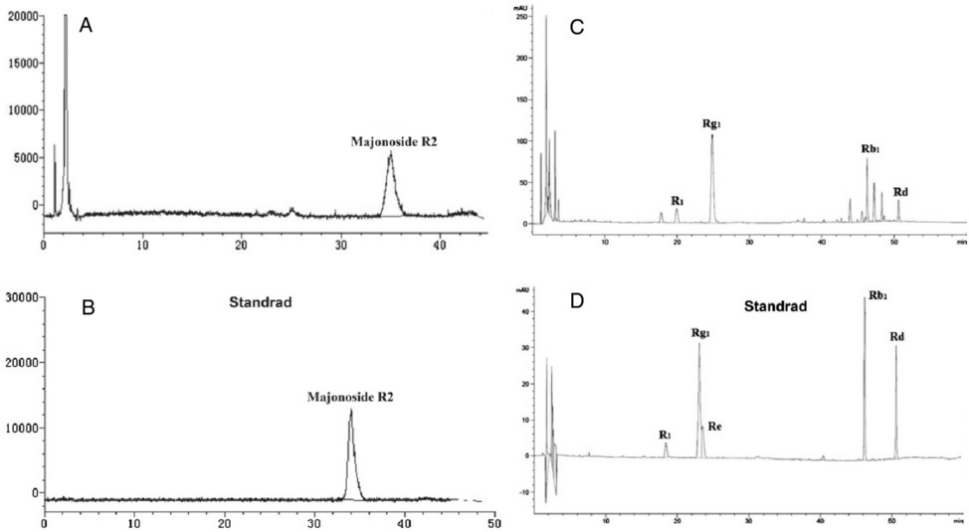
**Typical chromatograms of triterpenoid saponins in roots.** Typical chromatograms of triterpenoid saponins in *P. vietnamensis* var. *fuscidiscus* roots. **(A)** HPLC-ELSD chromatograms of majonoside R2 in *P. vietnamensis* var. *fuscidiscus* roots; **(B)** HPLC-ELSD chromatograms of authentic majonoside R2. **(C)** HPLC chromatograms of ginsenoside Rg1, Rb1, notoginsenoside R1, and ginsenoside Rd in *P. vietnamensis* var. *fuscidiscus* roots. **(D)** HPLC chromatograms of ginsenoside Rg1, Rb1, notoginsenoside R1, and ginsenoside standards.

The content of main component is the most widely used indicator to measure the quality of herb, so quantitative analysis of main component has important practical significance. According to previous research [2], majonoside R2, ginsenoside Rg1, Rb1, Rd and notoginsenoside R1 are considered as the five main components of *P. vietnamensis* var. *fuscidicus*. Herein, the content of five major triterpene saponins in roots of *P. vietnamensis* var. *fuscidicus* was determined. High performance liquid chromatography with evaporative light scattering detector (HPLC-ELSD) was employed for quantitative analysis of majonoside R2, due to its low UV absorptivity. As shown in Figure 10A and B, the peak of majonoside R2 was identified by direct comparing the retention times of the peaks with those of the standard majonoside R2 eluted under the same conditions. The content of majonoside R2 in in roots of *P. vietnamensis* var. *fuscidicus* is about 68 mg/g, indicated that majonoside R2 were rich in the roots of this species. Quantitative analysis of other four triterpene saponins in the roots of this herb were performed using high performance liquid chromatography (HPLC). As shown in Figure 10C and D, the investigated saponins were well separated within 55 min. The content of ginsenoside Rg1, Rb1, notoginsenoside R1, and ginsenoside Rd in the roots of this herb were approximately 52.7, 17.9, 17.8 and 3.2 mg/g, respectively. The above results were approximately in accordance with previous studies [2], indicated that our quantitative results are reliable. We believe that these data will be useful for pharmacological evaluation and quality control of this new variety.

## Conclusions

*P. vietnamensis* var. *fuscidiscus* exhibited remarkable disease resistance, and contains higher levels of ocotillol-type saponins. Thus, *P. vietnamensis* var. *fuscidiscus* is a suitable material for the study of ocotillol-type saponins biosynthesis and improvements of *Panax* plants. Because of the fact that *P. vietnamensis* var. *fuscidiscus* is a newly discovered variety of *P. vietnamensis*, no genomic information was available for this species. This is the first study performed on transcriptome sequencing of *P. vietnamensis* var. *fuscidiscus* using Illumina next-generation sequencing. In total, 126,758 unigenes were obtained. The large number of transcripts provided in this study not only facilitates the study of ocotillol-type saponins biosynthesis but also could provide opportunities to engineer microorganisms for the *de novo* production of active ingredients. Furthermore, numerous SSRs were identified and will be very useful for marker-assisted selection breeding of this herb.

## Methods

### Ethics statement

No specific permits were required for the described field studies. No specific permissions were required for these locations and activities. The location is not privately-owned or protected in any way and the field studies did not involve endangered or protected species.

### Plant material

Four-year-old *P. vietnamensis* var. *fuscidiscus* plants were collected from Jinping County, Yunnan province, southwest of China (Latitude: 22° 47′ 38″N, Longitude: 103° 2′ 22″E, Altitude: 1690 m), in May 2013. After morphological and molecular identification according the reference [24], the root tissues samples were collected separately from four randomly selected plant individuals. All samples were separately cut into small pieces, and parts of each sample were mixed with equivalent fresh weight (2 g) for RNA isolation. The remaining materials were used for *SE* gene cloning and RT-qPCR analysis. All samples were frozen immediately in liquid nitrogen and stored at −80°C until use.

### RNA library construction and sequencing

The total RNA was extracted from the mixed sample by using Trizol reagent (Invitrogen, Camarillo, CA, USA), following RNA purification by RNeasy MiniElute Cleanup Kit (Qiagen, Hilden, Germany), according to the manufacture’s protocol. The RIN (RNA integrity number) values of the isolated RNA were determined by using Agilent 2100 Bioanalyzer (Santa Clara, CA, USA). Samples with RIN of more than 8 were used for further analysis. The construction of the libraries and the RNA-Seq were performed by CapitalBio Corporation (Beijing, China). Firstly, poly (A) mRNA was purified from 20 μg of total RNA using Oligo(dT) magnetic beads. Then, mRNA was fragmented into smaller pieces (200–700 bp), which were used for first-strand cDNA synthesis with reverse transcriptase and random hexamer-primer. The second-strand cDNA was synthesized using buffer, dNTPs, RNaseH and DNA polymerase. The short double-stranded cDNA fragments are purified with QiaQuick PCR extraction kit (Qiagen, Hilden, Germany) and resolved with EB buffer. These cDNA fragments underwent an end-repair process and poly(A) was added and then ligated with the Illumina paired-end sequencing adaptors. Subsequently, Ligation products were purified with magnetic beads and separated by agarose gel electrophoresis. A range of cDNA fragments (200 ± 25 bp) were excised from the gel and selected for PCR amplification as templates. The cDNA library was constructed with a fragment-length range of 200 bp (±25 bp). Final, the cDNA library was sequenced on a paried-end flow cell using Illumina HiSeq™ 2000 platform.

### Transcriptome data processing and assembly

Before assembly, raw reads with adaptors and unknown nucleotides above 5% or those that were of low quality (containing more than 50% bases with Q-value ≤ 20) were removed to obtain clean reads using a custom Perl script. Then the clean reads were *de novo* assembled using Trinity program [60] with default parameters. First, clean reads with a certain length of overlap were combined to form longer fragments without N, which were called contigs. These clean reads were then mapped back to corresponding contigs with paired-end reads to detect contigs from the same transcript as well as the distances between contigs, and their paired-end information was also used to fill gaps or to extend the sequences. Finally, these resultant sequences were clustered to remove redundant sequences using the TIGR gene Indices clustering tools (TGICL) [61] to form longer sequences without N and cannot be extended on either end. Such sequences are defined as unigenes.

### Functional annotation and prediction of CDS

Functional annotations were performed by sequence comparison with public databases included the NCBI non-redundant nucleotide database (NT, by June 2012), non-redundant protein database (NR, by June 2012) (http://www.ncbi.nlm.nih.gov/), Swiss-Prot database (http://www.expasy.ch/sprot) and the Clusters of Orthologous Groups database (http://www.ncbi.nlm.nih.gov/COG/) [62] using BLASTN and BLASTX (http://blast.ncbi.nlm.nih.gov/Blast.cgi), respectively, with an e-value of 1e^−5^. A Perl script was written to assign the functional class to unigenes. Unigenes were also compared with KEGG [63] using BLASTX at an e-values of less than 1e^−10^. A Perl script was used to retrieve KEGG Orthology (KO) information from blast result and then established pathway associations between unigenes and database. Based on the results of Nr database annotation, we use Blast2GO program [64] to perform GO annotation of unigenes. After acheiving GO annotation for every unigene, WEGO [65] software was used to perform GO classification and to draw GO tree. Moreover, the conserved domains/families of the assembled unigenes encoding proteins were searched against the Pfam database (version 26.0) [66] using Pfam_Scan script.

The coding sequence (CDS) for unigene was predicted by BlastX and ESTscan. The unigene sequences were searched against the Nr, COG, KEGG and Swiss-Prot protein databases using BLASTX (e-value <10^−5^). Unigenes aligned to a higher priority database will not be aligned to lower priority database. The best alignment results were used to determine the sequence direction of unigenes. When a unigene could not be aligned to any database, ESTScan [67] program was used to predict coding regions and determine sequence direction.

### SSR detection and primer design

Potential SSR markers were detected among the 126,758 unigenes using the MISA tool (http://pgrc.ipk-gatersleben.de/misa/). We searched for SSRs with motifs ranging from mono- to hexa-nucleotides in size. The minimum of repeat units were set as follows: ten repeat units for mono-nucleotide, six for di-nucleotides, and five for tri-, tetra-, penta- and hexa-nucelotides. Primer pairs were designed using Primer3 (http://bioinfo.ut.ee/primer3-0.4.0/primer3/) with default parameters.

### Survey of SSR polymorphism

A total of 30 primer pairs (Additional file 17) were randomly selected to evaluate their application and the polymorphism across 13 *P. vietnamensis* var. *fuscidiscus* accessions (Additional file 18). Total DNA was isolated from *P. vietnamensis* var. *fuscidiscus* leaves using the CTAB method. PCR amplifications were conducted in a final volume of 20 μL containing 1 μL 2.5 mM dNTPs, 1 μL *EasyTaq* DNA polymerase (Beijing TransGen Biotech Co., Ltd. China), 2 μL 10 × *EasyTaq* buffer, 1 μL of each primer (10 μM), 13 μL ddH_2_O, and 1 μL template DNA (approx. 10 ng/μL). PCR was performed as follows: initial denaturation at 94°C for 2 min, followed by 35 cycles of denaturation for 30 s at 94°C, annealing for 30 s at different Tm depending on the gene, extension for 30 s at 72°C, and a final step of elongation at 72°C for 5 min. The separation of alleles was performed on 8% polyacrylamide gel. PCR products were mixed with an equal volume of loading buffer. The mixture was denatured at 95°C for 5 min before loading onto the gel.

### Data collection and analysis

The presence of each single band was coded as 1 and its absence as 0 in a data matrix. Base on the binary data matrix, popgene program version 1.32 [68] was use to calculate genetic variation parameters, including observed number of alleles (*No*), effective number of alleles (*Ne*), Shannon’s information index (*I*), number of polymorphic loci (*NP*) and percentage of polymorphic loci (*PPB*). Allelic data were used to calculate the polymorphism Information Content (PIC) of each SSR marker by using the formula: PIC =1–∑pi^2^ (pi is the frequency of i^th^ allele for each locus) [69]. By NTSYS pc 2.1 program [70], Jaccard's genetic similarity coefficients were calculated and dendrogram of the 13 *P. vietnamensis* var. *fuscidiscus* accessions was constructed by the UPGMA (un-weighted pair group method with arithmetic mean) clustering method.

### Full-length cDNA cloning of putative SE genes

Total RNA was reverse transcribed to synthesize first strand cDNA using oligo dT primer and a PrimeScript^TM^II 1st Strand cDNA Synthesis Kit (TaKaRa, Dalian, China) according to the manufacturer’s instructions. The RT-PCR products were used as template for cloning of PvfSE1, PvfSE2 and PvfSE3. The full-length cDNA sequences of PvfSE1, PvfSE2 and PvfSE3 were obtained from our transcriptome data. The specific primers (Additional file 19) used for the amplification of these genes were designed using primer3 program based on based on the predicted cDNA sequences and were then synthesized. PCRs were conducted in a total reaction volume of 25 μL, containing 1 μL of cDNA, 0.5 μM of each of the forward and reverse primers, 200 μM of dNTPs, 5 μL of 5 × Q5 Reaction Buffer, and 0.25 μL of Q5 High-Fidelity DNA polymerase (NEB, Beijing, China). The PCR conditions are as follows: 94°C for 3 min, followed by 35 cycles of 94°C for 1 min, 59°C for 1 min, 72°C for 5 min, with a final 10 min extention at 72°C. The PCR products were electrophoretically separatedon a 1% agarose gel, ligation into the pMD19-T vector (TaKaRa, Dalian, China) and were then subjected to automated DNA sequencing using the ABI 3730XL sequencer(Applied Biosystems, Foster City, USA).

### Phylogenetic analysis

Phylogenetic analysis was performed based on the deduced amino acid sequences of Cytochrome P450 (CYP450) and UDP-glycosyltransferase (UGT) from *P. vietnamensis* var. *fuscidiscus* and other plants. All of the deduced amino acid sequences were aligned with Clustal X using the default parameters: gap opening penalty, 10; gap extension penalty, 0.1; and delay divergent cutoff, 25%, and evolutionary distances were computed using MEGA5.10 with the Poisson correction method. For the phylogenetic analysis, a neighbor-joining tree was constructed using MEGA5.0. Bootstrap values obtained after 1000 replications are indicated on the branches. The scale repesents 0.1 amino acid substitutions per site.

### RT-qPCR analysis

Ten unigenes with potential roles in ginsenoside biosynthesis were chosen for validation using RT-qPCR with gene specific primers designed with Primer3 software. All the primers sequences used for the RT-qPCR analysis are shown in Additional file 20. Total RNA from different organs (roots, hairy roots, stems and leaves) of *P. vietnamensis* var. *fuscidiscus* were extracted individually using Trizol Kit (Promega, USA) following the manufacturer’s protocol. Subsequently, RNA was treated with 4 × gDNA wiperMix at 42°C for 2 min to remove DNA. The purified RNA (1ug) was reverse transcribed to cDNA using HiScript QRT SuperMix for qPCR (Vazyme, Nanjing, China). The qPCR reactions were performed in a 20 μl volume composed of 2 μl of cDNA, 0.4 μl of each primer, and 10 μl 2 × SYBR Green Master mix (TaKaRa) in Roche LightCycler 2.0 system (Roche Applied Science, Branford, CT). PCR amplification was performed under the following conditions: 30 s at 94°C, followed by 45 cycles of 94°C for 20 s, 55°C for 20 s, and 72°C for 30 s. Three technical replications were performed for all quantitative PCRs. The phosphomevalonate kinase (PMK) gene, which was found in our transcriptome database, was chosen as reference gene control for normalization after the expressions of three reference genes (actin, GAPDH, and PMK) were compared in different tissues. The relative changes in gene expression levels were calculated using the 2^-△△Ct^ method.

### HPLC- ELSD analysis of majonoside R2

The dried powder of *P. vietnamensis* var. *fuscidiscus* roots (0.11 g) were extracted by sonication with 50 ml of methanol for 45 min, let cool, then weighed and the weight of methanol to complement the weight loss, shake, with 0.45 μm microporous membrane filtration, and 10 μL of filtrate was analyzed by HPLC-ELSD. For majonoside R2 determination, a Shimadzu LC 20A HPLC system (Shimadzu, Kyoto, Japan) with a Sedex 75 evaporative light scattering detector (Sedere, Alfortville, France) was used. Chromatographic separation was performed on an Waters symmetry shield™RP_10_ (4.6 mm × 250 mm, 5.0 um, Milford, MA, USA) column maintained at 30°C. The mobile phase was acetonitrile-water (19.5:80.5, v/v), and the flow rate was 1 μL/min. The drift tube temperature of ELSD was set at 40°C and nebulizer nitrogen gas flow-rate was 1.5 l/min and gain of 9. Authentic majonoside R2 was provided by Yunnan Institute for Food and Drug Control (Kunming, Yunnan, China).

### HPLC analysis of other four triterpene saponins

In brief, the dried powder of *P. vietnamensis* var. *fuscidiscus* roots (0.6 g) were extracted with 40 mL of 100% MeOH for 30 min and sonicated for 60 min and then diluted to 50 mL with MeOH. The methanol extract was filtered through a 0.45 μm membrane filter and 10 μL of filtrate was directly injected into the HPLC system.

Quantitative analysis of the remaining four triterpene saponins (ginsenoside Rg1, Rb1, notoginsenoside R1, and ginsenoside Rd) in the roots of this herb was performed on Agilent 1260 HPLC systems (Agilent Technologies, Santa Clara, CA, USA). The chromatographic column Agilent Zorbar SB-C_18_ (250 mm × 4.6 mm, 5 μm, Agilent Technologies, Santa Clara, CA, USA) was used and the column temperature was maintained at 30 °C. The flow rate was fixed at 1 mL/min, and the mobile phase consisted of acetonitrile (A) and water (B) and separation was achieved using the following gradient system: 85% B at 0 min, 80% B at 5 min, 77% B at 30 min, 60% B at 50 min, and 60% B at 55 min. Detection was performed at 203 nm for the remaining four triterpene saponins. Authentic ginsenoside Rg1, Rb1, notoginsenoside R1, and ginsenoside Rd were purchased from J&K Scientific Ltd (Beijing, PR China).

## Additional files

Additional file 1:
**Assessment of assembly quality.**
Additional file 2:
**Comparison of P. vietnamensis var. fuscidiscus unigenes to orthologous P. notoginseng coding sequences.**
Additional file 3:
**Venn diagram results from diverse databases.**
Additional file 4:
**Comparison of unigene length between hit and no hit unigenes.**
Additional file 5:
**Characterization of searching the assembled unigenes against NCBI Nr and Swiss-Prot protein databases. **
Additional file 6: Top-hit species distribution for sequences from *P. vienamensis* var. *fuscidiscus* submitted BLASTX against the NCBI-Nr database. Additional file 7:
**Gene Ontology classification.**
Additional file 8: List of Pfam domain families assigned to *P. vietnamensis* var. fuscidiscus unigenes. Additional file 9: Top 20 Pfam domainsfamilies predcted in P. vienamensis var. *fuscidiscus*. Additional file 10: Mapping of *P.vietnamensis* var. *fuscidiscus* unique sequences to KEGG biochemical pathways. Additional file 11: Information of SSR derived from all unigene. Additional file 12: Sequence informations of SSR primers. Additional file 13: Characteristics of 15 polymorphic EST-SSR primer pairs in 13 *P. vietnamensis* var. *fuscidiscus* accessions. Additional file 14: The main identified triterpene saponin biosynthetic genes from *P. vietnamensis* var. *fuscidiscus* unigenes. Additional file 15: Cytochrome P450 discovery. Additional file 16: The main identified glycosyltransferase genes from *P. vietnamensis* var. *fuscidicus* unigenes. Additional file 17: SSR primer pairs validated in this study. Additional file 18:
*P. vietnamensis* var. *fuscidiscus* germplasms for polymorphism validation with EST-SSRs. Additional file 19: Gene-specific used for reverse-transcriptase PCR assays. Additional file 20: Primers used for gene expression analysis by reverse transcription quantitative real-time PCR (RT-qPCR).

## Abbreviations

NCBI: National Center for Biotechnology Information
Nr: Non-redundant protein
COG: Cluster of Orthologous Groups
GO: Gene Ontology
KEGG: Kyoto Encyclopedia of Genes and Genomes
SSRs: Simple sequence repeats
cDNA: Complementary DNA
BLAST: Basic Local Alignment Search Tool
CDS: Coding sequence
bp: Base pair
AACT: Acetyl-CoA acetyltransferase
β-AS: β-amyrin synthase
DMAPP: Dimethylallyl diphosphate
DS: Dammarenediol-II synthase
FPP: Farnesyl diphosphate
FPPS: Farnesyl diphosphate synthase
Glc: Glucose
GPP: Geranyl pyrophosphate
GGPP: Geranylgeranyl diphosphate
GGPPS: Geranylgeranyl pyrophosphate synthase
GT: Glycosyltransferase
HMG-CoA: 3-hydroxy-3-methylglutaryl coenzyme A
HMGR: HMG-CoA reductase
HMGS: HMG-CoA synthase
IPP: Isopentenyl diphosphate
IPPI: IPP isomerase
MVD: Mevalonate diphosphate decarboxylase
MVK: Mevalonate kinase
P450: Cytochrome P450
PMK: Phosphomevalonate kinase
SE: Squalene epoxidase
SS: Squalene synthase
RT-PCR: Reverse transcription PCR
RT-qPCR: Reverse transcription quantitative real-time PCR
HPLC-ELSD: High performance liquid chromatography with evaporative light scattering detector
HPLC: High performance liquid chromatography
