# Supplementary material for: Transcriptome analysis of Panax vietnamensis var. fuscidicus discovers putative ocotillol-type ginsenosides biosynthesis genes and genetic markers
Source: BMC Genomics. 2015 Mar 8;16(1):159. doi: 10.1186/s12864-015-1332-8 (PMC4355973; doi:10.1186/s12864-015-1332-8)
Supplement: Additional file 1: — Assessment of assembly quality. [file 12864_2015_1332_MOESM1_ESM.docx]

**Additional File 1. Assessment of assembly quality.** Distribution of unique-mapped reads of the assembled unigenes.

**
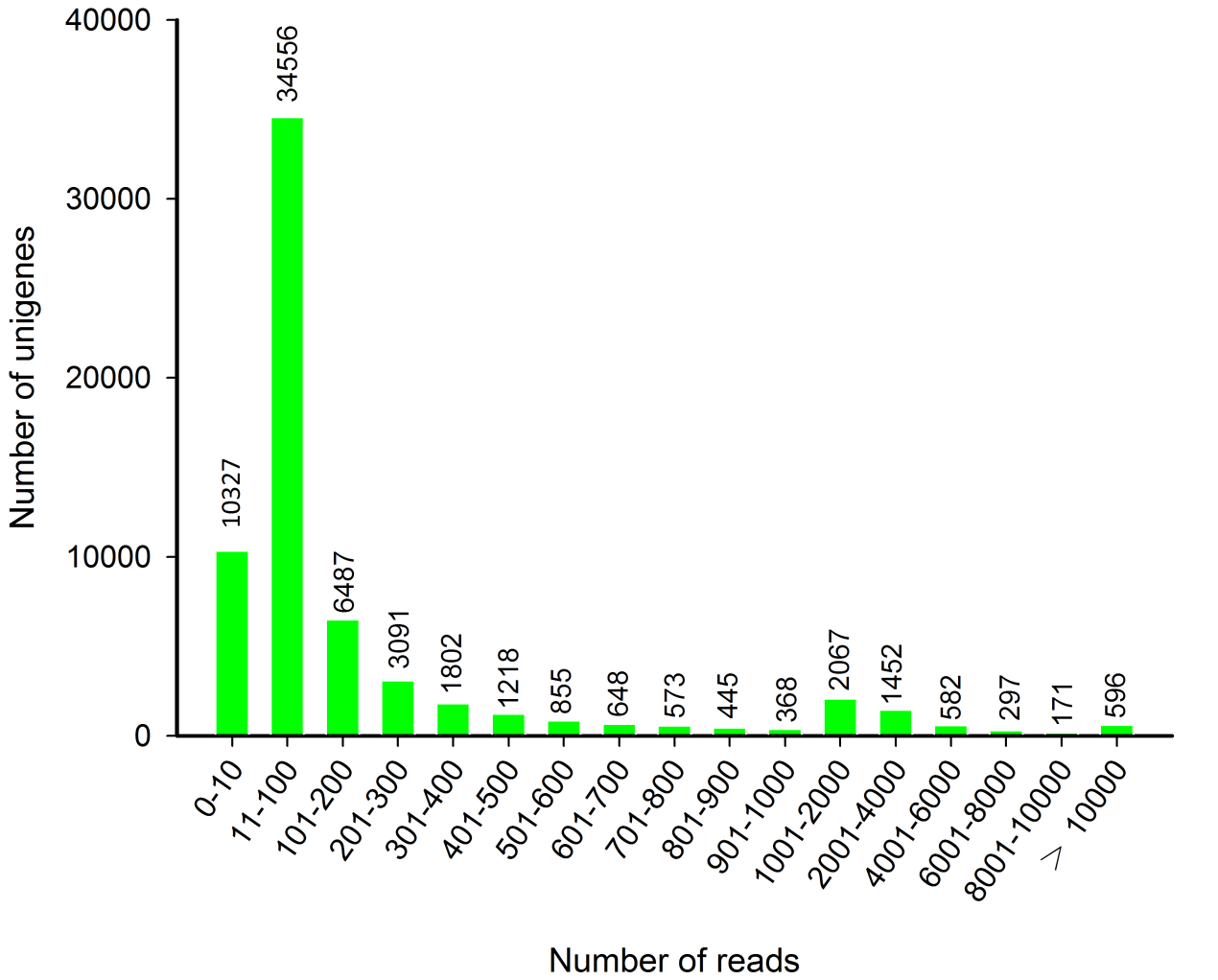
**
