# Supplementary material for: Transcriptome analysis of Panax vietnamensis var. fuscidicus discovers putative ocotillol-type ginsenosides biosynthesis genes and genetic markers
Source: BMC Genomics. 2015 Mar 8;16(1):159. doi: 10.1186/s12864-015-1332-8 (PMC4355973; doi:10.1186/s12864-015-1332-8)

**Additional File 2.** **Comparison of** ***P. vietnamensis var. fuscidiscus* unigenes to orthologous *P. notoginseng* coding sequences.** (A) The ratio of *P. vietnamensis var. fuscidiscus* unigene length to *P. notoginseng* ortholog length was plotted against *P. vietnamensis var. fuscidiscus* unigene coverage depth. (B) Total percent of *P. notoginseng* ortholog coding sequence that was covered by all *P. vietnamensis var. fuscidiscus* unigenes.


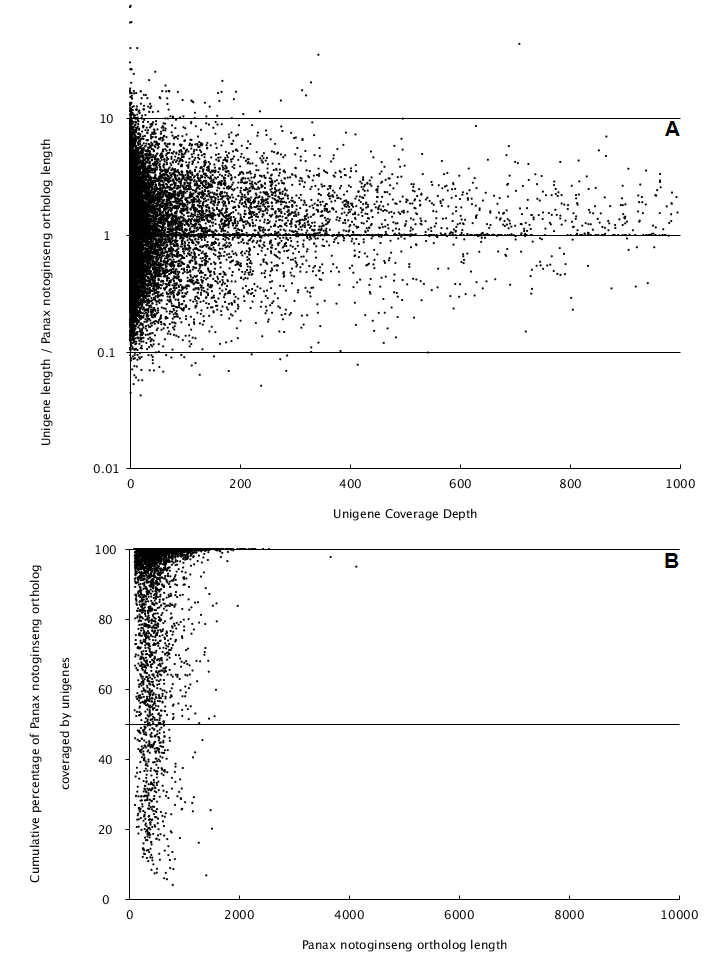

Supplement: Additional file 2: — Comparison of P. vietnamensis var. fuscidiscus unigenes to orthologous P. notoginseng coding sequences. [file 12864_2015_1332_MOESM2_ESM.docx]
