# Supplementary material for: Transcriptome analysis of Panax vietnamensis var. fuscidicus discovers putative ocotillol-type ginsenosides biosynthesis genes and genetic markers
Source: BMC Genomics. 2015 Mar 8;16(1):159. doi: 10.1186/s12864-015-1332-8 (PMC4355973; doi:10.1186/s12864-015-1332-8)
Supplement: Additional file 3: — Venn diagram results from diverse databases. [file 12864_2015_1332_MOESM3_ESM.docx]

**Additional file 3. Venn diagram results from diverse databases.** Venn diagram of number of unigenes annotated by BLASTX with an E-value threshold of 10^-5^ against protein databases. The numbers in the circles indicate the number of unigenes annotated by single or multiple databases.

**
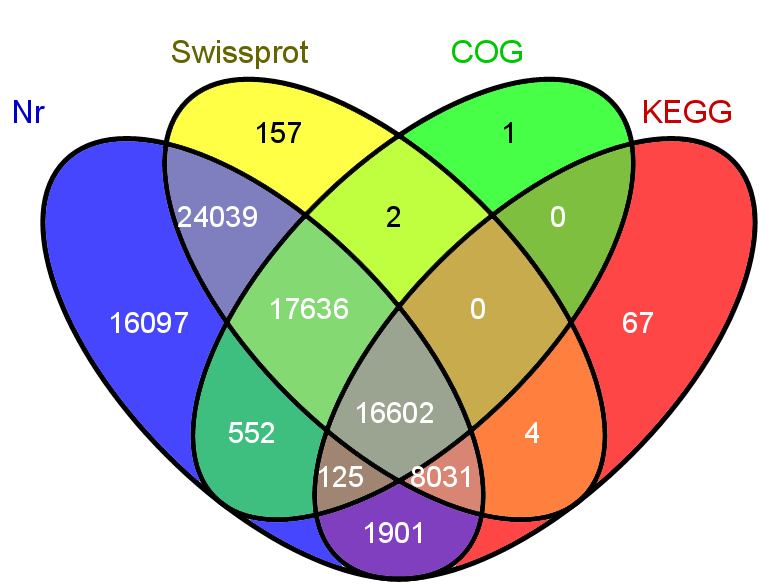
**
