# Supplementary material for: Transcriptome analysis of Panax vietnamensis var. fuscidicus discovers putative ocotillol-type ginsenosides biosynthesis genes and genetic markers
Source: BMC Genomics. 2015 Mar 8;16(1):159. doi: 10.1186/s12864-015-1332-8 (PMC4355973; doi:10.1186/s12864-015-1332-8)

**Additional file 4. Comparison of unigene length between hit and no hit unigenes.** **(A)** Comparison of unigene length between hit and no hit unigenes in the Nr databases. **(B)** Comparison of unigene length between hit and no hit unigenes in the Swiss-prot database. Longer unigenes were more likely to have BLASTx homologs in protein database.


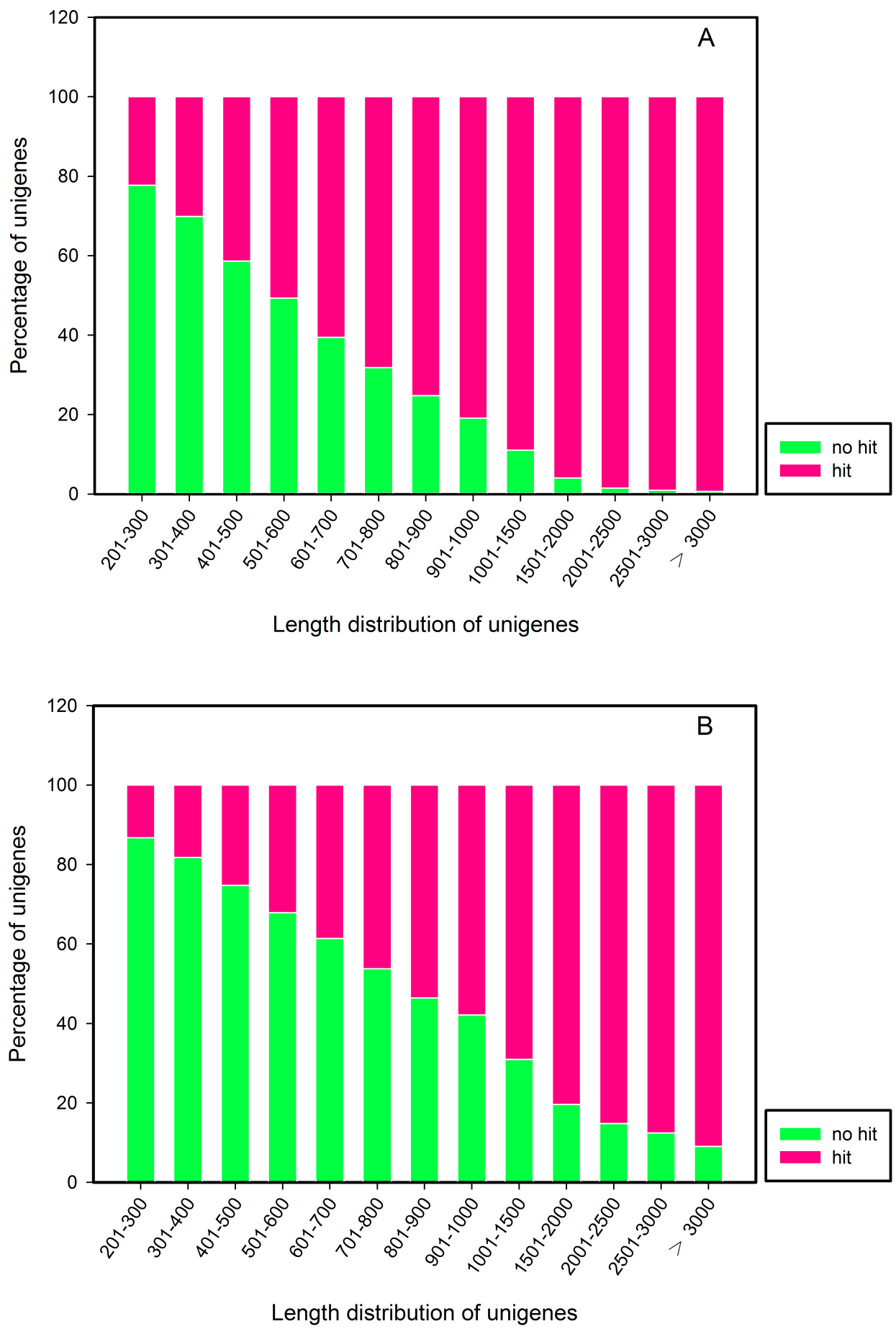

Supplement: Additional file 4: — Comparison of unigene length between hit and no hit unigenes. [file 12864_2015_1332_MOESM4_ESM.docx]
