# Supplementary material for: Transcriptome analysis of Panax vietnamensis var. fuscidicus discovers putative ocotillol-type ginsenosides biosynthesis genes and genetic markers
Source: BMC Genomics. 2015 Mar 8;16(1):159. doi: 10.1186/s12864-015-1332-8 (PMC4355973; doi:10.1186/s12864-015-1332-8)

**Additional file 5. Characterization of searching the assembled unigenes against NCBI Nr and Swiss-Prot protein databases.** **(A)** E-value proportional frequency distribution of BLAST hits against the Nr database. **(B)** E-value proportional frequency distribution of BLAST hits against the Swiss-Prot database. **(C)** Similarity distribution of the top BLAST hits for the assembled unigenes with a cutoff of 1E-5 in Nr database. **(D)** Similarity distribution of the top BLAST hits for the assembled unigenes with a cutoff of 1E-5 in Swiss-Prot database.


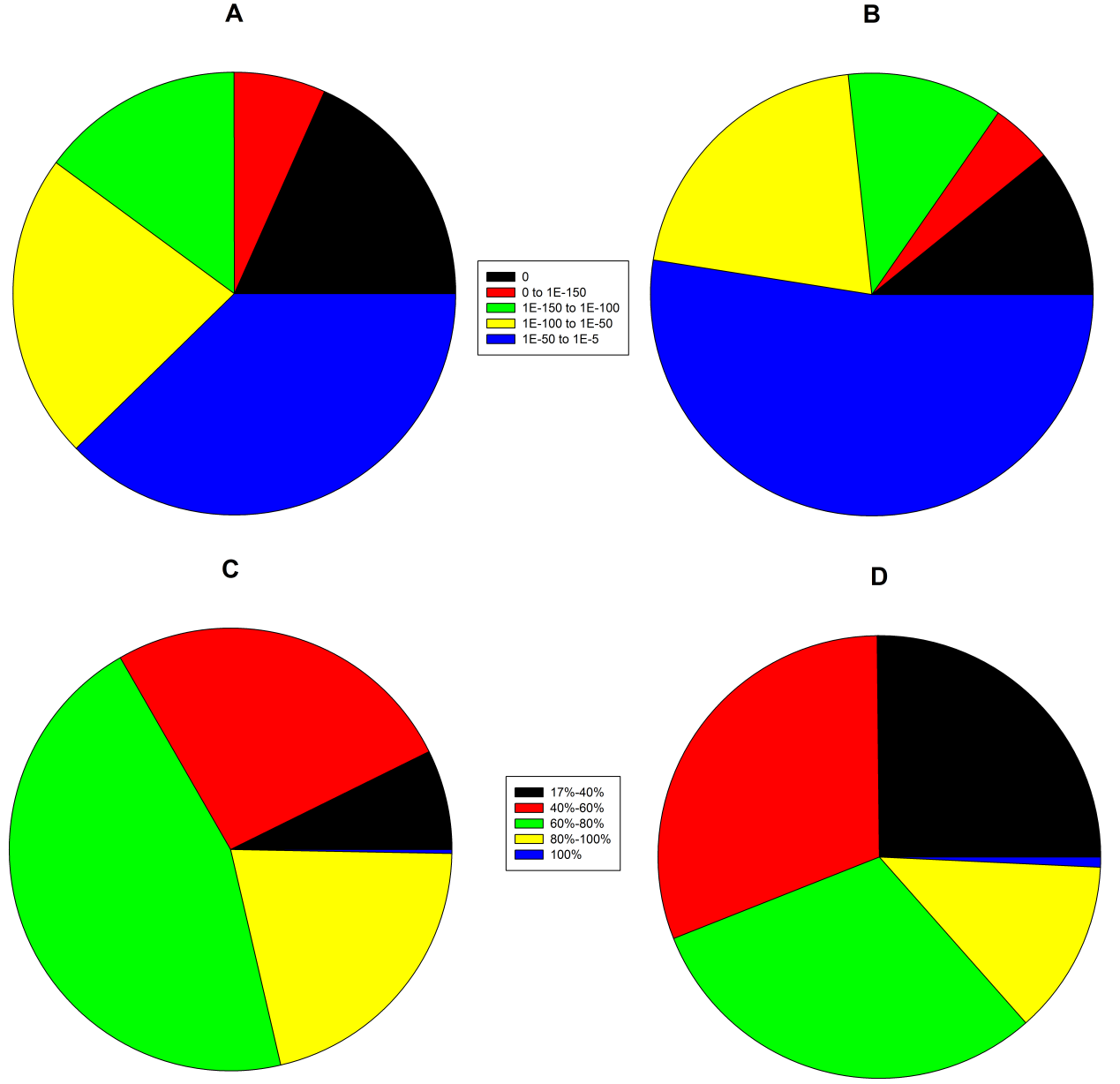

Supplement: Additional file 5: — Characterization of searching the assembled unigenes against NCBI Nr and Swiss-Prot protein databases. [file 12864_2015_1332_MOESM5_ESM.docx]
