# Supplementary material for: Transcriptome analysis of Panax vietnamensis var. fuscidicus discovers putative ocotillol-type ginsenosides biosynthesis genes and genetic markers
Source: BMC Genomics. 2015 Mar 8;16(1):159. doi: 10.1186/s12864-015-1332-8 (PMC4355973; doi:10.1186/s12864-015-1332-8)

**Additional file 6.** **Top-hit species distribution for sequences from *Panax vienamensis var. fuscidiscus* submitted BLASTX against the NCBI-Nr database.**


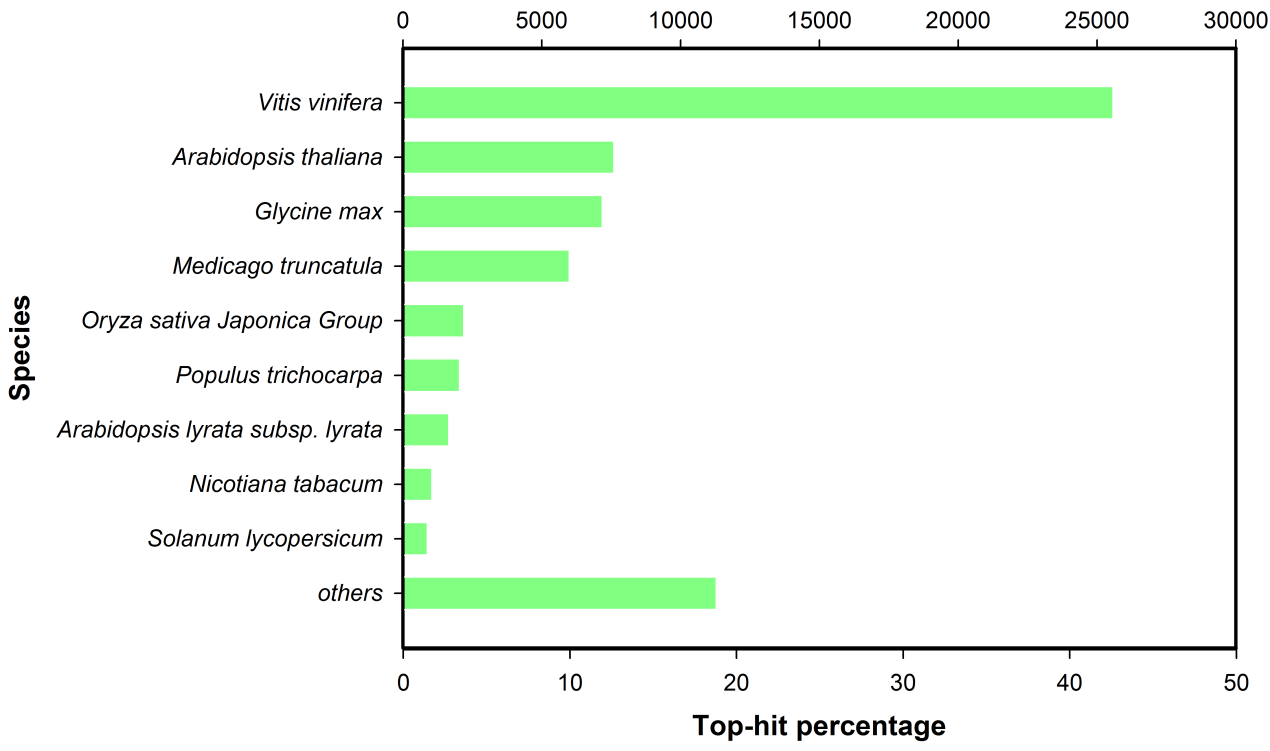

Supplement: Additional file 6: — Top-hit species distribution for sequences from P. vienamensis var. fuscidiscus submitted BLASTX against the NCBI-Nr database. [file 12864_2015_1332_MOESM6_ESM.docx]
