# Supplementary material for: Transcriptome analysis of Panax vietnamensis var. fuscidicus discovers putative ocotillol-type ginsenosides biosynthesis genes and genetic markers
Source: BMC Genomics. 2015 Mar 8;16(1):159. doi: 10.1186/s12864-015-1332-8 (PMC4355973; doi:10.1186/s12864-015-1332-8)
Supplement: Additional file 7: — Gene Ontology classification. [file 12864_2015_1332_MOESM7_ESM.docx]

**Additional file 7. Gene Ontology classification**

| Gene Ontology | Class | Total numbers |
| --- | --- | --- |
| Biological process | anatomical structure formation | 374 |
|  | biological adhesion | 7 |
|  | biological regulation | 3,648 |
|  | cell killing | 8 |
|  | cellular component biogenesis | 585 |
|  | cellular component organization | 2,444 |
|  | cellular process | 20,747 |
|  | death | 234 |
|  | developmental process | 3,828 |
|  | establishment of localization | 4,138 |
|  | growth | 470 |
|  | immune system process | 366 |
|  | localization | 4,248 |
|  | locomotion | 8 |
|  | metabolic process | 21,803 |
|  | multi-organism process | 815 |
|  | multicellular organismal process | 3,076 |
|  | pigmentation | 2,682 |
|  | reproduction | 1,741 |
|  | reproductive process | 1,729 |
|  | response to stimulus | 6,352 |
|  | rhythmic process | 127 |
|  | viral reproduction | 53 |
| Cellular component | cell | 28,886 |
|  | cell part | 28,886 |
|  | envelope | 1,632 |
|  | extracellular region | 308 |
|  | extracellular region part | 34 |
|  | macromolecular complex | 3,178 |
|  | membrane-enclosed lumen | 744 |
|  | organelle | 19,977 |
|  | organelle part | 5,339 |
| Molecular function | antioxidant activity | 178 |
|  | binding | 22,925 |
|  | catalytic activity | 22,836 |
|  | electron carrier activity | 13 |
|  | enzyme regulator activity | 310 |
|  | molecular transducer activity | 1,115 |
|  | structural molecule activity | 533 |
|  | transcription regulator activity | 480 |
|  | translation regulator activity | 512 |
|  | transporter activity | 2,640 |
