# Supplementary figures and images for: Transcriptome analysis of Panax vietnamensis var. fuscidicus discovers putative ocotillol-type ginsenosides biosynthesis genes and genetic markers
Source: BMC Genomics. 2015 Mar 8;16(1):159. doi: 10.1186/s12864-015-1332-8 (PMC4355973; doi:10.1186/s12864-015-1332-8)

**Additional file 9. Top 20 Pfam domains/families predcted in *P. vienamensis* var. *fuscidiscus*.**


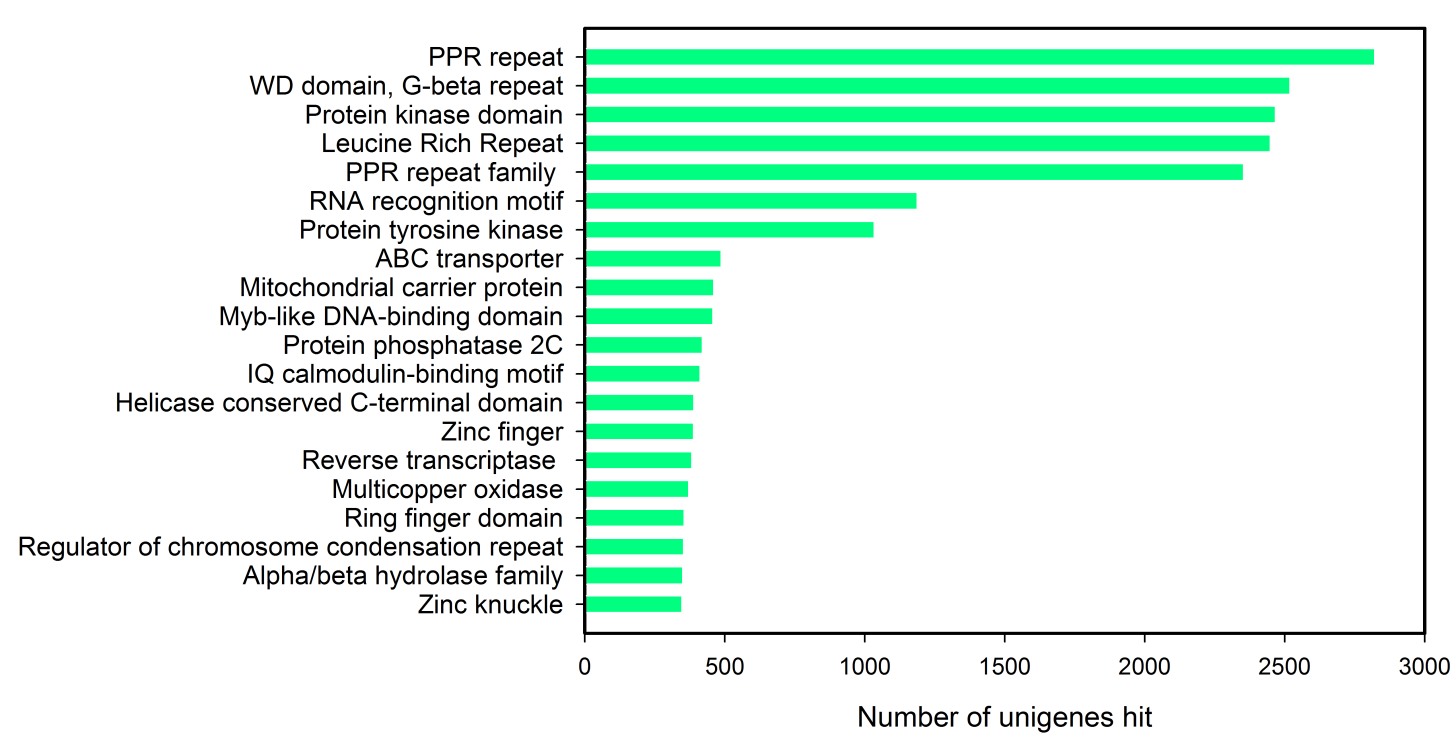

Supplement: Additional file 9: — Top 20 Pfam domainsfamilies predcted in P. vienamensis var. fuscidiscus. [file 12864_2015_1332_MOESM9_ESM.docx]
