# Supplementary material for: Transcriptome analysis of Panax vietnamensis var. fuscidicus discovers putative ocotillol-type ginsenosides biosynthesis genes and genetic markers
Source: BMC Genomics. 2015 Mar 8;16(1):159. doi: 10.1186/s12864-015-1332-8 (PMC4355973; doi:10.1186/s12864-015-1332-8)
Supplement: Additional file 10: — Mapping of P.vietnamensis var. fuscidiscus unique sequences to KEGG biochemical pathways. [file 12864_2015_1332_MOESM10_ESM.doc]

**Additional file 10. Mapping of *P.vietnamensis var.fuscidiscus* unique sequences to KEGG biochemical pathways.**

| **KEGG categories represented** | **No. of uniques** |
| --- | --- |
| **Metabolism** | **15,587** |
| Amino acid metabolism | 2,352 |
| Carbohydrate metabolism | 3,809 |
| Nucleotide metabolism | 1,137 |
| Lipid metabolism | 2,023 |
| Energy metabolism | 1,782 |
| Metabolism of cofactors and vitamins | 1,053 |
| Metabolism of other amino acids | 710 |
| Metabolism of terpenoids and polyketides | 497 |
| Glycan biosynthesis and metabolism | 1,282 |
| Biosynthesis of other secondary metabolites | 457 |
| **Genetic Information Processing** | **7,880** |
| Folding, Sorting, and Degradation | 2,486 |
| Replication and Repair | 1,200 |
| Transcription | 1,228 |
| Translation | 2,966 |
| **Environmental Information Processing** | **2,616** |
| Signal Transduction | 2,475 |
| Membrane Transport | 131 |
| Signaling Molecules and Interaction | 10 |
| **Cellular Processes** | **5473** |
| Cell Communication | 546 |
| Cell Growth and Death | 2,010 |
| Cell Motility | 271 |
| Transport and Catabolism | 1,743 |
| **Organismal Systems** | **5,483** |
| **Human Diseases** | **4,865** |
