# Supplementary material for: Transcriptome analysis of Panax vietnamensis var. fuscidicus discovers putative ocotillol-type ginsenosides biosynthesis genes and genetic markers
Source: BMC Genomics. 2015 Mar 8;16(1):159. doi: 10.1186/s12864-015-1332-8 (PMC4355973; doi:10.1186/s12864-015-1332-8)
Supplement: Additional file 13: — Characteristics of 15 polymorphic EST-SSR primer pairs in 13 P. vietnamensis var. fuscidiscus accessions. [file 12864_2015_1332_MOESM13_ESM.docx]

**Additional file S13.** Characteristics of 15 polymorphic EST-SSR primer pairs in 13 *P. vietnamensis* var. *fuscidiscus* accessions.

| **Primer** | **Forward primer (5’-3’)** | | **Reverse primer (5’-3’)** | ***No*** | ***Ne*** | ***I*** | ***NP*** | ***PPB*** | ***PIC*** |
| --- | --- | --- | --- | --- | --- | --- | --- | --- | --- |
| P01 | | GTTATCATGTAGTCCCTCAAACACC | TTTAGTCATGTGCCCTCCT | 0.33 | 0.27 | 0.21 | 1.6 | 17.78 | 0.47 |
| P07 | | GCAATGTGTAGGAGACTTTGTAACC | GCTTGCCAACTGCAGTAGATTT | 0.42 | 0.34 | 0.27 | 1.8 | 20.00 | 0.50 |
| P09 | | GCTGAATATGAAACGGAAATAGAGC | AAAGCTGCATAGACTGAAGGTTCTT | 0.25 | 0.20 | 0.16 | 1.2 | 13.33 | 0.49 |
| P12 | | CTGCACATACAAACAAGCAGCTAAT | TGGTTCTGTTTGCATTCTGT | 0.47 | 0.38 | 0.30 | 2.6 | 28.89 | 0.32 |
| P15 | | TGCTTATTCTGGACATGCAAAC | ACAACAAACAAGTCTCCTGTAGAGC | 0.38 | 0.31 | 0.24 | 2.2 | 24.44 | 0.29 |
| P18 | | CAAACAAGACATACCATAACAGAGG | ACTCATCAATCACCCAACTGTAGAT | 0.49 | 0.39 | 0.31 | 3.6 | 40.00 | 0.41 |
| P19 | | ATTCAATGCTATGAAGCACAACCAC | AAGGAAAGCCAGGGATGTCT | 0.39 | 0.31 | 0.25 | 2.2 | 24.44 | 0.46 |
| P24 | | CAAACCAGGCTGTTGTTGTTG | CATCATCATCAGATTCATCGCTAGT | 0.49 | 0.39 | 0.31 | 3.2 | 35.55 | 0.47 |
| P26 | | ATCCGGATGCTCCAAATC | GGGAAATAGATGGTCTCTAATAGC | 0.48 | 0.39 | 0.31 | 3.4 | 37.78 | 0.48 |
| P30 | | TAAACTTATGGTCTGGCGATAACC | GTGGAAGGCTTCAATCAGACTTTAT | 0.47 | 0.38 | 0.21 | 2.6 | 28.89 | 0.46 |
| P31 | | GGCGTCTTTGTGTGAAAGAACT | GAGCAAGGCAATCTACATTCTACAC | 0.33 | 0.27 | 0.21 | 1.4 | 15.55 | 0.50 |
| P36 | | CACTGAGAAGGAAGAACAGTATAAGG | AGATAAGAAATGGCTAACGGCTTTG | 0.49 | 0.39 | 0.31 | 3.0 | 33.33 | 0.49 |
| P41 | | CAGAAGCTGTTAATGTGATTAGCTC | TTCTCTACTAGCTGATGGATCCTGT | 0.27 | 0.21 | 0.17 | 0.8 | 8.89 | 0.38 |
| P43 | | ATAAAGTCTGAACCGGACTATAACG | TTCGGTAGTCACGCTTAACAAT | 0.50 | 0.40 | 0.32 | 4.4 | 48.89 | 0.50 |
| P50 | | TTGATTATACCTCCCTTACGTTGTC | CCATGCAAATAACACATACATGGTC | 0.52 | 0.41 | 0.33 | 4.2 | 46.67 | 0.46 |
| Mean | | |  | 0.42 | 0.34 | 0.27 | 2.5 | 28.15 | 0.44 |

*No*: Observed number of alleles; *Ne*: Expective number of alleles; *I*: Shannon’s information index; NP: Number of polymorphic loci; PPB: Percentage of polymorphic loci; PIC: Polymorphism Information Content.
