# Supplementary material for: Transcriptome analysis of Panax vietnamensis var. fuscidicus discovers putative ocotillol-type ginsenosides biosynthesis genes and genetic markers
Source: BMC Genomics. 2015 Mar 8;16(1):159. doi: 10.1186/s12864-015-1332-8 (PMC4355973; doi:10.1186/s12864-015-1332-8)
Supplement: Additional file 18: — P. vietnamensis var. fuscidiscus germplasms for polymorphism validation with EST-SSRs. [file 12864_2015_1332_MOESM18_ESM.docx]

**Additional file 18. *P. vietnamensis* var. *fuscidiscus* germplasms for polymorphism validation with EST-SSRs.**

| **Code** | **Source** |
| --- | --- |
| MX11068 | Jingping, Yunnan, China |
| MX11075 | Jingping, Yunnan, China |
| MX11082 | Jingping, Yunnan, China |
| YY1145 | Yuanyang, Yunnan, China |
| YY-2 | Yuanyang, Yunnan, China |
| YY-3 | Yuanyang, Yunnan, China |
| Laos1201 | Laos |
| Laos1203 | Laos |
| Laos-1 | Laos |
| ZM-1 | Jingping, Yunnan, China |
| ZM-2 | Jingping, Yunnan, China |
| TS1232 | Jingping, Yunnan, China |
| TS1239 | Jingping, Yunnan, China |
