# Supplementary material for: Transcriptome analysis of Panax vietnamensis var. fuscidicus discovers putative ocotillol-type ginsenosides biosynthesis genes and genetic markers
Source: BMC Genomics. 2015 Mar 8;16(1):159. doi: 10.1186/s12864-015-1332-8 (PMC4355973; doi:10.1186/s12864-015-1332-8)
Supplement: Additional file 19: — Gene-specific used for reverse-transcriptase PCR assays. [file 12864_2015_1332_MOESM19_ESM.docx]

Additional file 19. Gene-specific used for reverse-transcriptase PCR assays.

| Gene | Primer sequences |
| --- | --- |
| **PvfSE1** | **Forward: GTCGCTCGCATTGTTCATCA**  **Reverse: TCTGTGGAACGAGGATTGCA** |
| **PvfSE2** | **Forward: ATTCATGGAACTCGGGAGGA**  **Reverse: AGTTGTCTGTCTGCTCTTGAGA** |
| **PvfSE3** | **Forward: AACGTCCAACCAACACCATG**  **Reverse: TCGCCCGTGACTGATATTCA** |
